# A food web bioaccumulation model for the accumulation of per- and polyfluoroalkyl substances (PFAS) in fish: how important is renal elimination?†

**DOI:** 10.1039/d2em00047d

**Published:** 2022-05-25

**Authors:** Jennifer M. Sun, Barry C. Kelly, Frank A. P. C. Gobas, Elsie M. Sunderland

**Affiliations:** Harvard John A. Paulson School of Engineering and Applied Sciences, Harvard University Cambridge MA USA 02138 jennifersun@.g.harvard.edu; Meta Analytical Inc. Calgary AB T3H 2Z5 Canada; School of Resource and Environmental Management, Faculty of the Environment, Simon Fraser University Burnaby British Columbia V5A 1S6 Canada

## Abstract

Per- and polyfluoroalkyl substances (PFAS) are a large class of highly fluorinated anthropogenic chemicals. Some PFAS bioaccumulate in aquatic food webs, thereby posing risks for seafood consumers. Existing models for persistent organic pollutants (POPs) perform poorly for ionizable PFAS. Here we adapt a well-established food web bioaccumulation model for neutral POPs to predict the bioaccumulation behavior of six perfluoroalkyl acids (PFAAs) and two perfluoroalkyl ether acids (HFPO-DA, 9-Cl-PF3ONS) produced as PFAA replacements. The new model includes sorption to blood plasma proteins and phospholipids, empirically parameterized membrane transport, and renal elimination for PFAAs. Improved performance relative to prior models without these updates is shown by comparing simulations to field and lab measurements. PFAS with eight or more perfluorinated carbons (*η*_pfc_ ≥ 8, *i.e.*, C8 perfluorosulfonic acid, C10–C11 perfluorocarboxylic acid, 9-Cl-PF3ONS) are often the most abundant in aquatic food webs. The new model reproduces their observed bioaccumulation potential within a factor of two for >80% of fish species, indicating its readiness to support development of fish consumption advisories for these compounds. Results suggest bioaccumulation of *η*_pfc_ ≥ 8 PFAS is primarily driven by phospholipid partitioning, and that renal elimination is negligible for these compounds. However, specific protein binding mechanisms are important for reproducing the observed tissue concentrations of many shorter-chain PFAAs, including protein transporter-mediated renal elimination. Additional data on protein-binding and membrane transport mechanisms for PFAS are needed to better understand the biological behavior of shorter-chain PFAAs and their alternatives.

## Abstract 2

We present a whole-body aquatic food web bioaccumulation model for perfluoroalkyl acids and two alternatives. Results highlight structure-dependent mechanisms, including phospholipid partitioning, blood plasma protein binding, and renal elimination.

## Graphical abstract

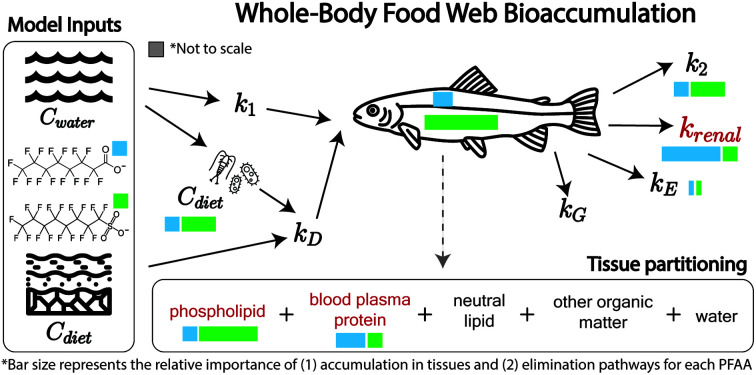

**Environmental significance**

Exposures to PFAS have been linked to adverse health effects for both humans and wildlife. Seafood ingestion is an important human exposure source for PFAS, and bioaccumulation models are needed to support risk assessments and the development of fish consumption advisories. Here, we present a new mechanistic aquatic food web bioaccumulation model for simulating concentrations of ionizable PFAS in fish. The model successfully reproduces observed average whole body fish tissue concentrations within a factor of 2 for the most bioaccumulative perfluoroalkyl acids and their alternatives. Model results highlight structure-dependent bioaccumulation patterns, including the importance of specific protein binding mechanisms for the accumulation and elimination of PFAS with <8 perfluorinated carbons.

## Introduction

Per- and polyfluoroalkyl substances (PFAS) are a large class of persistent anthropogenic chemicals widely used by industry and in consumer products since the 1950s.^1,2^ Exposures to a few well-studied perfluoroalkyl acids (PFAAs) have been statistically associated with many adverse health effects in epidemiological studies.^3,4^ Observed PFAA concentrations in human blood are often correlated with reported seafood consumption.^5,6^ Fish and other seafood are the primary dietary source of perfluorooctane sulfonate (C8 PFSA or PFOS) for adults in the general European population, and a main dietary source of the C8 PFSA and perfluoroalkyl carboxylates (PFCAs) with eight or more perfluorinated carbons (*η*_pfc_ ≥ 8) in the United States (U.S.).^7,8^ Fish consumption advisories for PFAS have been implemented in several U.S. states and are being developed in others.^9–13^ Food-web bioaccumulation models can provide helpful decision-support tools for such guidelines.

Mechanistic food web models that predict tissue concentrations of toxicants based on measurements in water and sediment have been widely used to assess the bioaccumulation potential of neutral and lipophilic persistent organic chemicals (POPs). A key theoretical assumption of these models is that bioaccumulation is driven by equilibrium partitioning between the exposure medium and organism tissues. For neutral POPs, the sorption capacity of an organism is approximated based on partitioning to neutral storage lipids.^14,15^ In contrast, tissue distributions of PFAAs and PFAA alternatives with highly acidic functional groups are controlled by both phospholipids and proteins.^16–20^ In addition, some PFAAs are eliminated *via* facilitated transport pathways across tissue membranes.^21–23^

The relative importance of partitioning to different tissue types varies across PFAAs.^17,19,24^ Limited data are available for the C8 PFSA and C8 PFCA on kinetic processes such as organic anion transporter-mediated renal elimination in aquatic organisms.^25,26^ Bioconcentration models that are based on either phospholipid sorption or protein binding show different strengths when compared to whole-body concentrations or tissue-specific distribution patterns in fish.^16,18,19,27^ For example, Dassuncao *et al.*^20^ found that concentrations of long-chain PFAAs in marine mammal organs varied based on their phospholipid content. PFAA accumulation in fish appears to linearly increase with longer carbon chain-length up to 11 perfluorinated carbons and then declines.^28,29^ This behavior may be better explained by similar curvilinear chain-length patterns observed in tissue-specific binding proteins and renal elimination proteins.^19^ Both phospholipid partitioning and protein-binding mechanisms, including active transport pathways, are therefore important for predicting PFAA bioaccumulation.^17,19^

Dietary ingestion is understood to be an important uptake pathway for many PFAAs.^30–33^ However, only aqueous uptake has been included in prior mechanistic PFAA bioaccumulation models for fish.^16,18^ Modifications to existing models are thus needed before such tools can be reliably used to predict accumulation in aquatic food webs.

Here, we present a new mechanistic food web model for PFAAs and ionizable PFAA alternatives in aquatic ecosystems, modified from a widely used bioaccumulation model for POPs. The model includes tissue partitioning to specific binding proteins and phospholipids, as well as renal elimination, among other updates. We evaluate the model using previously published lab and field datasets for six PFAAs and two PFAA alternatives. The utility of mechanistic bioaccumulation models for informing regulatory and risk evaluations for PFAS is discussed based on this assessment.

## Methods

### Model overview

**Fig. 1:**
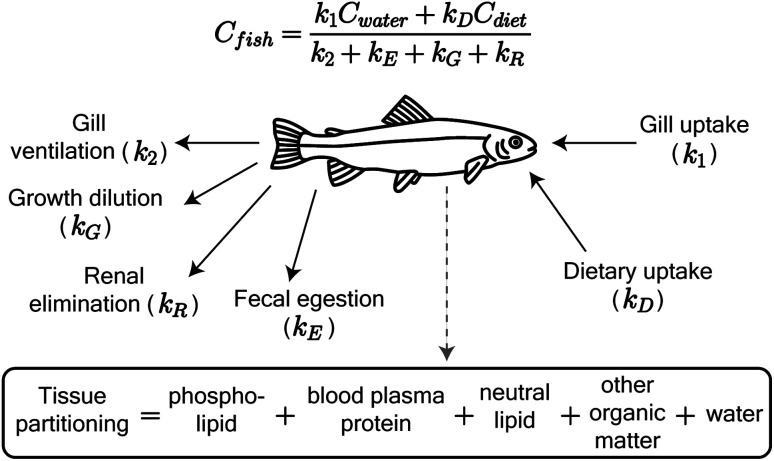
Schematic of the PFAA model in fish, including uptake and elimination pathways and tissue partitioning compartments. Partitioning to fish tissues drives the balance between uptake and passive elimination (gill ventilation and fecal egestion) pathways. Renal elimination is parameterized using chain-length dependent patterns in renal clearance-to-reabsorption ratios and renal-to-branchial elimination ratios (ESI Section 8†). The invertebrate model includes the same uptake pathways but omits renal elimination.

We adapted the Arnot and Gobas^15^ bioaccumulation model for neutral, hydrophobic POPs in aquatic food webs to predict whole-body tissue concentrations of PFAAs (Fig. 1). The original model included chemical uptake through respiration and diet, and chemical loss through respiration, growth dilution, and fecal egestion. Armitage *et al.*^16^ previously adapted the same model to describe the bioconcentration of ionizable organic compounds in fish by including partitioning to phospholipids and accounting for pH-dependent chemical transport across membranes. We build on this model here by including: (1) partitioning to both phospholipids and binding proteins, (2) a new empirically parameterized renal elimination pathway for fish, (3) updated model parameters for chemical absorption, and (4) a full food web simulation.

We simulated the whole-organism food web bioaccumulation of six PFAAs because parameterization and evaluation data were readily available for these compounds: perfluorosulfonic acids (PFSAs) with six perfluorinated carbons (C6) and eight carbons (C8); perfluorocarboxylic acids (PFCAs) with eight to eleven carbons (C8–C11). We did not extend the model to other PFAAs because controlled laboratory data for model evaluation were not available. We additionally extended the bioconcentration model to perfluoroalkyl ether acids, a class of PFAA replacement compounds that has been widely detected in the environment.^34,35^ We evaluated one short chained compound, HFPO-DA (hexafluoropropylene oxide-dimer acid, the major component of GenX), and one long-chained compound, 9-Cl-PF3ONS (9-chlorohexadecafluoro-3-oxanone-1-sulfonic acid, the major component of F53B), for which partitioning and evaluation data were available.

A full description of the model algorithms and parameters is provided in the ESI (Sections 1–6†). Many PFAAs are known to preferentially accumulate in the kidney and liver of wildlife.^24^ The model developed here is most suitable for assessing exposures of fish consumers rather than wildlife toxicity risk assessments because we did not include organ-specific accumulation. For large fish, literature-reported muscle-to-whole body conversion factors can be used to translate modeled whole-body concentrations to muscle fillet concentrations relevant to human exposure.^36^

### Tissue partitioning

Five tissue phases were considered in the partitioning model: neutral lipids, phospholipids, blood plasma binding proteins (albumin or albumin-like proteins), non-lipid organic matter, and water. Partitioning to neutral lipids, non-lipid organic matter, and water follows the parameterization developed by Armitage *et al.*^16^ for ionizable organic compounds. We used empirical partition coefficients for phospholipids to model membrane-water distribution ratios^37,38^ (Tables S2, S3a, S6a and b†). Sorption to blood plasma proteins was based on empirical protein-water partition coefficients in human serum albumin^39^ (Tables S2, S3b, S6a and b†). Liver fatty acid binding proteins (L-FABP) are thought to contribute to elevated liver PFAS concentrations in fish and other organisms.^27,40,41^ We find on a whole-body basis in fish partitioning to L-FABP is negligible compared to blood plasma protein partitioning and therefore neglect it in this model^18,42^ (ESI Sections 3 and 7†).

### Respiratory uptake

Chemical uptake by phytoplankton was described using a two-phase resistance model^15^ that we adapted to consider the membrane-water partition coefficient (Table S1†). Respiratory uptake by invertebrates and fish was based on the ventilation rate of each specific organism and chemical absorption efficiency across gill membranes (Tables S1, S2, S6a and b†). We derived chemical absorption efficiencies from water using fish bioconcentration studies^27,43,44^ (ESI Section 3, Tables S6a and b†). No data were available for aquatic invertebrates, so we used the fish values to estimate uptake.

At environmentally relevant pH, prior work has shown that passive diffusion of PFAAs through lipid bilayers is pH-independent for PFSAs and only weakly pH-dependent for PFCAs^38^ (ESI Section 3†). For PFCAs, transport of the neutral species through the interior of lipid bilayer membranes is orders of magnitude greater than that of the ionic species. As a result, transport of the neutral PFCA fraction remains relevant despite the extremely small size of the neutral fraction at biologically relevant pH. For PFSAs, the neutral fraction is less important due to greater permeability of the ionic fraction compared to PFCAs. This may reflect the lower surface charge densities and broader surface charge distribution in the larger sulfonate headgroup compared to the carboxylate headgroup.^38^ Paracellular transport, ion channel transport, and/or protein-mediated transport pathways also typically become more important than pH-dependent diffusive transport as chemical ionization increases for large, charged molecules.^16,45,46^ We therefore approximate gill membrane transport as pH-independent across a range of environmentally relevant conditions and use empirically derived absorption efficiency parameters (Tables S2, S6a and b; ESI Section 3†).

### Dietary uptake

Dietary uptake of PFAAs for invertebrates and fish was calculated based on prey consumption rates and the chemical absorption efficiency across gut membranes (Tables S1 and S2†). Absorption efficiencies were derived from uptake rates measured in fish biomagnification studies. These studies showed that absorption efficiencies increase with fluorinated chain length and are greater for PFSAs compared to PFCAs of the same chain length.^41,47^ Lower dietary absorption rates were observed in adults compared to juveniles, so we parameterized adult and juvenile fish with different values (ESI Section 3†).

### Elimination pathways

The amount of chemical partitioned to tissues determines the ratio between uptake and passive elimination rates (respiratory and fecal), following the Arnot and Gobas^15^ model (Table S1†). Chemical absorption efficiencies are higher with greater PFAA chain length, which causes increases in both uptake and elimination rates as PFAA chain length increases. Chemical partitioning to tissues also increases with PFAA chain length and is greater for PFSAs compared to PFCAs. As a result, respiratory and fecal elimination rates increase more slowly and non-linearly than uptake rates as PFAA chain-length grows (Table S1†).

Growth dilution was modeled using previously published empirical relationships between growth and both temperature and organism size.^15^ PFAAs and the two PFAA alternatives evaluated are not known to degrade *in vivo*, so chemical loss through metabolism was not included in the model.

Substantial renal elimination of PFAAs has been observed for some organisms. Prior *in vivo* studies show decreasing renal clearance rates with increasing chain lengths for the C6–C10 PFCAs in monkeys and rats,^23^ and for PFSA in rats.^48^ To parameterize renal elimination in a fish bioconcentration model for PFAAs, Ng and Hungerbuhler^18^ used data from *in vitro* studies that showed the same pattern of decreasing net renal secretion with increasing fluorinated carbon chain length. Their analysis was conducted using renal clearance and reabsorption rate constants calculated from uptake rates measured in cells expressing various rat organic anion transporters.^22^ Clearance rates increased more slowly than reabsorption rates with increasing fluorinated chain length for the C7–C10 PFCAs (*i.e.*, clearance-to-reabsorption rate ratio decreased from 5.9 to 1.2).

Based on these findings, we included an empirical renal elimination parameterization for PFAAs in fish. Our parameterization is based on *in vivo* rainbow trout renal elimination rates for the C8 PFCA and C8 PFSA,^25,26^ and the renal clearance-to-reabsorption ratios reported in prior work.^18,22^ Renal clearance-to-reabsorption ratios for PFSAs were not measured directly in the *in vitro* studies, so we used ratios estimated from the PFCA with the same number of perfluorinated carbons.^18^ We first calculated renal elimination rates for the C8 PFCA and C8 PFSA in juvenile rainbow trout measured in laboratory-controlled bioconcentration and biomagnification studies,^27,41^ using the ratios of renal to branchial elimination measured in two separate studies of adult rainbow trout^25,26^ (Table S8†). Renal elimination in these studies was inferred to be primarily driven by active protein transport mechanisms. Elimination through glomerular filtration was thought to be negligible because the C8 PFCA was measured to be nearly 100% bound in the blood, and similar bound fractions are expected for other PFAAs. We assumed the C12 PFCA renal elimination rate was zero, which is consistent with the linear extrapolation of the renal clearance-to-reabsorption ratios for the C7–C10 PFCAs from Ng and Hungerbuhler.^18,22^ To estimate renal elimination rates for PFAAs without measured values, we assumed a linear relationship between the fish renal elimination rates calculated for the C8 PFSA, C8 PFCA, and C12 PFCA (*i.e.*, zero) and their renal clearance-to-resorption ratios (Fig. S8†).

We then calculated renal-to-branchial elimination ratios for all PFAAs, using branchial elimination rates modeled for the laboratory-controlled study fish. Branchial elimination rates were calculated as a function of the aqueous uptake rate (Table S1; ESI Section 3,† see “chemical absorption efficiencies”), which was measured for all compounds except the C9 PFCA. We used a linear regression between fluorinated carbon chain length and aqueous uptake rate to interpolate parameterization data for the C9 PFCA.^27^ Renal elimination rates for the model application to field study fish were then calculated using the renal-to-branchial elimination ratios calculated from these laboratory-controlled studies.

### Data synthesis and model evaluation

We synthesized two main data sets that were useful for PFAA model evaluation. The first was laboratory data on bioconcentration and biomagnification of PFAAs^27,41^ that maintained exposure at constant levels, and provided data on organism weight, growth rates, and chemical uptake rates. The availability of these measured parameters allowed us to evaluate the tissue partitioning and renal elimination parametrizations while minimizing other uncertainties. Model performance was quantified by comparing mean modeled and observed bioconcentration factors (BCFs) and biomagnification factors (BMFs).

The second dataset consisted of field observations from the Gironde Estuary, France.^33^ We modeled six fish (goby [*Pomatoschistus* spp.], anchovy [*Engraulis encrasicolus*], sprat [*Sprattus sprattus*], sole [*Solea solea*], flounder [*Platichthys flesus*], common seabass [*Dicentrarchus labrax*]) and five invertebrate (ragworm [*Nereis diversicolor*], *Copepoda*, *Mysidacea*, *Gammarus* spp., white shrimp [*Palaemon longirostris*]) species. These species represent a benthic food web (sole, flounder) and a benthopelagic food web that includes both small pelagic fish species (anchovy, sprat) and species with mixed diet and habitat use (goby, common seabass). For invertebrates, whole organism PFAA concentrations were directly measured. For fish species, both whole-body and muscle tissue PFAA concentrations were analyzed for two species, while only muscle tissue concentrations were reported for others. The authors of this study used measured muscle-to-whole body concentration ratios to convert to whole-body tissue concentrations for all fish species.^33^ We examined the central tendency and ranges of measured water, sediment and biota PFAA concentrations to evaluate modeling results. For water and sediment, raw data were not reported so we estimated the median as the midpoint between the reported minimum and maximum reported values. Individual sample measurements were provided for biota and used to calculate median values. Values below detection were represented as the 
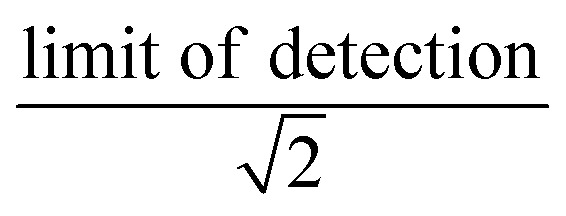
. For each PFAA, species with <50% detectable values were excluded from the model.

To evaluate specific model mechanisms or parameterizations, model results for individual species were first assessed using observed rather than modeled prey concentrations to minimize potential error propagation. Food web relationships and most environmental parameters were estimated from prior work in the Gironde Estuary.^33,49–51^ To evaluate performance across the full food web, we then modeled dietary PFAS concentrations using the estimated median of the measured water and sediment concentrations. Differences between modeled and field-reported median whole-body tissue concentrations across species and/or PFAAs were used to quantify (1) model bias and (2) absolute model bias for PFAA *j* and species *i*, where *C*_model_ is the model-predicted tissue PFAA concentration, *C*_obs_ is the observed tissue PFAA concentration, and *n* is the number of samples. A PFAA predicted with MB = 2 can be interpreted as being on average a factor of two overpredicted. A model prediction with an AMB < 2 can be interpreted as being within a factor of two of the observed value.1
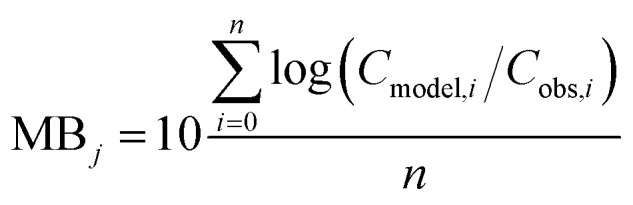
2
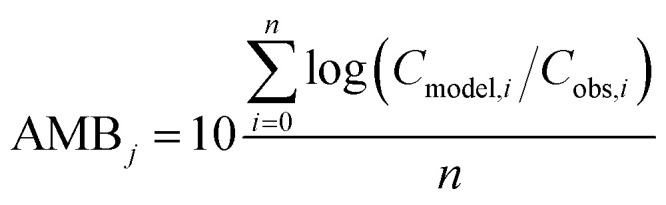


For the PFAA alternatives, model bias was quantified by comparing mean modeled and observed bioconcentration factors. Model evaluation data were only available from two laboratory bioconcentration studies.^43,44^ Dietary exposure was not considered in these studies and therefore is not included in our model application for these compounds. Modeled fecal egestion was extremely low (<0.1% of total elimination) for PFAAs in the juvenile rainbow trout BCF study, in large part due to small fish size and low dietary consumption rates. The alternative PFAA BCF studies followed a similar study design and therefore we expect similarly low rates of fecal egestion. We therefore exclude fecal elimination from the BCF models for the alternative PFAAs.

### Sensitivity analysis

We identified parameters that had the greatest impact on modeled fish tissue concentrations by perturbing each parameter by 10% and evaluating the corresponding change in modeled tissue concentrations. These results were used to a calculate a sensitivity ratio (
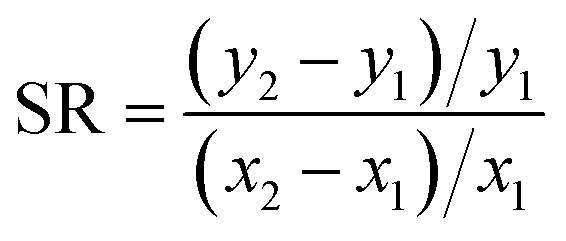
, where *x* is the modeled tissue concentration and *y* is the model parameter). Several modeled variables representing key biological processes or chemical-specific mechanisms (*i.e.*, feeding rate, ventilation rate, aqueous chemical absorption efficiency) were calculated from organism and chemical parameters such as organism weight, dissolved oxygen concentration, or partition coefficients, and directly assessed in the sensitivity analysis.

## Results and discussion

### Evaluation of fish model with renal elimination

**Fig. 2:**
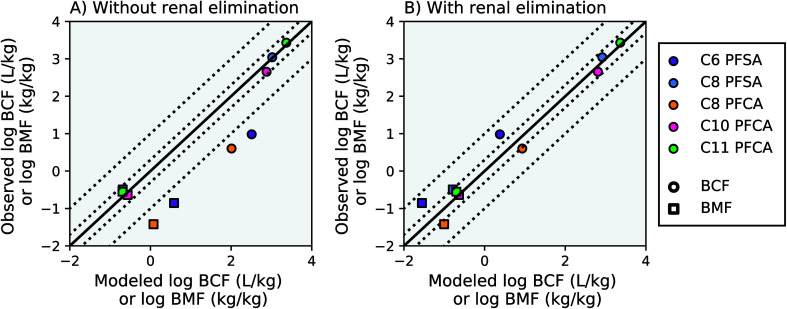
Modeled and observed mean PFAA bioconcentration factors (BCF) and biomagnification factors (BMF) based on laboratory data for juvenile rainbow trout.^27,41^ The solid black line represents a 1 : 1 line (perfect agreement) between observed and modeled results, while the dotted lines represent a factor of two and a factor of 10 difference between modeled and observed values. Panel (A) shows model results without renal elimination and panel (B) shows results with renal elimination, which is a new process added to the model. The C9 PFCA was used as an internal standard in the laboratory study and therefore is not shown.

Modeling results from this study agree within a factor of two of the observed laboratory BCF and BMF measurements for the C8 PFSA and C10–C11 PFCAs, with or without the algorithm for renal elimination (Fig. 2, AMB < 2). For PFAAs with fewer than 8 perfluorinated carbons (*η*_pfc_ < 8), model results exceed observations by more than an order of magnitude without the addition of an algorithm for renal elimination (Fig. 2A). The addition of a renal elimination algorithm reduces absolute model bias to less than three for the C8 PFCA (PFOA), and less than five for the C6 PFSA in both the BCF and BMF models (Fig. 2B). Renal elimination minimally affects fish body burdens for the more bioaccumulative compounds (C8 PFSA and C10–C11 PFCAs) but helps to explain accumulation patterns for PFAA with *η*_pfc_ < 8 in fish. Renal elimination for PFSAs was parameterized using protein binding data for PFCAs of the same perfluorinated chain length. Reasonable model performance based on this parameterization suggests that chain-length may play a more important role than head group for renal elimination in fish.

For the PFAAs with *η*_pfc_ < 8, the important role of renal elimination is likely due in part to the extremely low respiratory elimination rates characteristic of these weakly hydrophobic and highly ionized compounds (Table S2, ESI Section 3†). Chemical absorption efficiencies (*E*_W_) estimated from aqueous uptake rates for these compounds are orders of magnitude lower than that of neutral POPs. As a result, although the volume of water ventilated through the gills is several orders of magnitude greater than the volume of urinary excretion,^18^ the effective respiratory clearance rate (*E*_W_ multiplied by gill ventilation) is still comparable to observed renal clearance rates in fish. As PFAA chain length, and hydrophobicity, increases, chemical absorption efficiencies approach the same order of magnitude as that of neutral POPs^15,16,27^ (*i.e.* C11 PFCA, ESI Section 3†). The overall bioaccumulation behavior of the more hydrophobic PFAAs therefore also approaches that of neutral POPs, for which respiratory elimination is orders of magnitude greater and renal elimination is of negligible importance.

These patterns have been observed in limited laboratory studies of adult rainbow trout. In two studies used to inform the parameterization of renal-to-branchial elimination ratios in this model, the C8 PFCA (log *K*_OW,N_ = 5.30, seven perfluorinated carbons) contributed about 80% of total (renal + branchial) elimination, compared to 20% for the C8 PFSA (log *K*_OW,N_ = 6.43, eight perfluorinated carbons) (Fig. S8†). Renal elimination for the C8 PFCA was approximately three times that of the C8 PFSA.^25,26^ This difference in magnitude likely reflects patterns in relative renal elimination protein binding strengths. For example, terrestrial animal studies^18,22^ show an increasing role for renal elimination with decreasing PFAA carbon chain length for both PFCAs and PFSAs. Together, these analyses suggest that renal elimination mechanisms in terrestrial animals may be similarly relevant in fish. Additional laboratory data are needed to further characterize and directly quantify the renal elimination pathway across PFAS for aquatic species, including further assessment of the effects of fluorinated carbon tail length and functional headgroup on renal elimination rates.

### Food web model evaluation

**Fig. 3:**
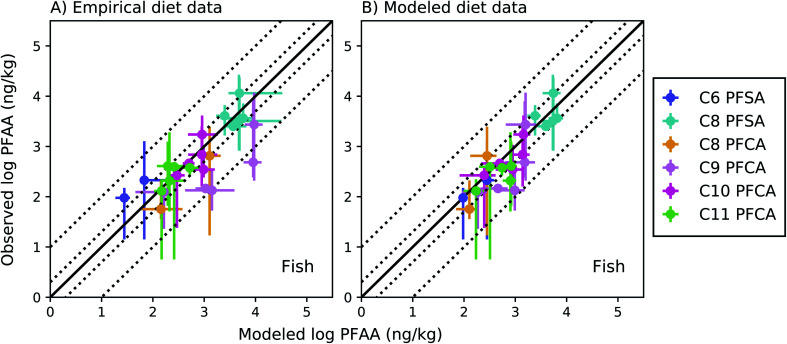
Field-evaluation of PFAA bioaccumulation model for fish from the Gironde Estuary, France,^33^ simulated using observed prey data (A) and modeled prey values (B). The solid black line represents a 1 : 1 line (perfect agreement), while the dotted lines represent a factor of two and a factor of 10 difference between modeled and observed values. Circles show modeled values based on median water/sediment/prey concentrations and error bars represent minimum and maximum reported exposure concentrations. Model error in (A) primarily reflects model performance for individual fish species, while model error in (B) includes error propagated from lower trophic levels. In general, modeled variability is lower than observed, and is lower in (B) than (A) due to error propagation. Observed variability likely includes additional variability not included in the model, such as variability in organism or environmental parameters.

The full food web simulation considered aqueous and dietary uptake together for both fish and invertebrates. Simulations based on empirical (measured rather than modeled) prey concentrations produced modeled average whole-body fish tissue concentrations that fell within a factor of 2 of observed medians for the C8 PFSA and C10–C11 PFCAs, and within a factor of 3.5 for the PFAAs with fewer than 8 perfluorinated carbons (C6 PFSA AMB = 3.3; C8 PFCA AMB = 2.2) (Fig. 3a). Greater error for the C9 PFCA (AMB = 5.7) compared to other PFAAs likely reflects uncertainty in the aqueous chemical absorption efficiency, which was interpolated based on chain-length dependent patterns rather than empirically measured (ESI Section 3†). For the food web simulation based on modeled (rather than measured) prey concentrations, average whole-body fish tissue concentrations fell within a factor of 3 for all PFAAs (Fig. 3b).

For the C8 PFSA and C10–C11 PFCAs, 80% of fish values modeled using measured prey concentrations fell within a factor of two, and all fell within a factor of three, of observed medians across concentration ranges spanning two orders of magnitude (empirical prey *R*^2^ = 0.83; simulated prey *R*^2^ = 0.71). For the C6 PFSA and C8 PFCA, whole-body median concentrations fell within a factor of 3.5 of observed medians for individual species, and always within the range of observed values (Fig. 3a). For the C9 PFCA, modeled values fell within a factor of 1.3–18 of observed medians likely due to parameter uncertainty, as discussed above.

**Fig. 4:**
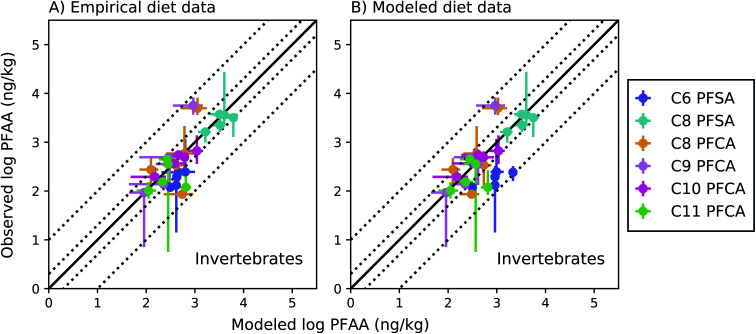
Field-evaluation of PFAA bioaccumulation model for invertebrates from the Gironde Estuary, France.^33^ Results are simulated using observed prey data^33^ (A) and modeled prey values (B). The solid black line represents a 1 : 1 line (perfect agreement), while the dotted lines represent a factor of two and a factor of 10 difference between modeled and observed values. Circles show modeled values based on median water/sediment/prey concentrations and error bars represent minimum and maximum reported exposure concentrations. Model error in (A) primarily reflects model performance for individual invertebrate species, while model error in (B) includes error propagated from lower trophic levels. In general, modeled variability is lower than observed, and is lower in (B) than (A) due to error propagation. Observed variability likely includes variability not included in the model, such as variability in organism or environmental parameters.

Among invertebrates, absolute model bias averaged across species ranged from 1.3 to 2.4 for each PFAA in the empirical prey model (Fig. 4a). Median ragworm concentrations were underestimated by a factor of 4 and 5 for the C8 and C9 PFCAs and overestimated by a factor of 6 for the C11 PFCA. This is consistent with prior lab observations that showed an inverse relationship between PFAA carbon chain length and concentrations in oligochaetes. This result likely reflects higher sorption of the long chain PFAAs to sediment solids, resulting in reduced bioavailability in the gut.^52^ Similar chain-length patterns were observed for other benthic invertebrates in the Gironde Estuary. Chain-length dependent partitioning of PFAAs to sediment solids may thus affect bioavailability more broadly for benthic species.

Results of our model evaluation compare well with previous studies. Modeled whole-body fish concentrations from the multi-compartment PBTK model based on protein-binding mechanisms developed by Ng and Hungerbuhler^18^ underestimated observed values by an order of magnitude or more for all PFAAs except the C8 PFCA. Results from the phospholipid-based mass balance model for ionizable chemicals developed by Armitage *et al.*^16^ fell outside an order of magnitude error for at least one of the modeled PFAAs (C8–C11 PFCAs and C8 PFSA) in alternate model versions using different phospholipid partitioning coefficients. Inclusion of both phospholipid and protein-binding mechanisms for PFAAs improved the performance of the model developed in this study compared to these prior PFAA bioconcentration models, indicating that both mechanisms should be considered in future applications.

### Partitioning to tissue matrices

**Fig. 5:**
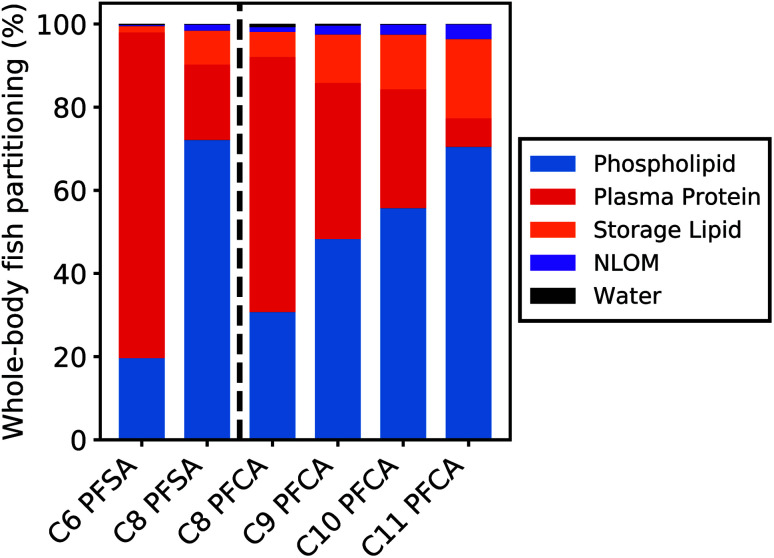
Modeled whole-body partitioning of PFAAs in fish to five tissue types: phospholipids, blood plasma proteins, neutral storage lipids, non-lipid organic matter (NLOM) such as carbohydrates and structural proteins, and water. Less than 1% of the total chemical mass is estimated to remain unbound in the aqueous phase (water).

Fig. 5 shows the relative contribution of each tissue matrix to whole-body partitioning across PFAAs (see equation for *D*_bw_, Table S1†). Modeled whole-body fish tissue sorption is dominated by phospholipids for the C8 PFSA and C11 PFCAs (>70%). In contrast, blood plasma proteins are the primary driver of accumulation for the C6 PFSA (>70%). The relative contribution of phospholipids to whole-body partitioning increases with carbon chain length for both PFCAs and PFSAs. This is consistent with prior work that showed significant correlations between PFAS and phospholipid concentration for only the long-chained PFAAs, and greater increases in PFAA concentration for a given increase in phospholipid content with increasing PFAA chain length.^20^ Conversely, the relative contribution of blood plasma proteins decreases with carbon chain length, contributing less than 30% of whole-body partitioning for the C8 PFSA and C9–C11 PFCAs.

For both PFCAs and PFSAs, the strength of partitioning increases linearly and more rapidly across chain lengths for phospholipids compared to proteins. This may reflect greater hydrophobic tail-group interactions in phospholipid partitioning compared to protein binding.^53,54^ This chain-length dependent pattern in tissue partitioning is particularly pronounced between the C6 and C8 PFSAs. Blood plasma protein partitioning strengths for these two PFSAs are comparable, unlike PFCAs of the same chain-length difference, while phospholipid partitioning strengths increase more log-linearly across chain lengths for both PFCAs and PFSAs. Accordingly, model results show that both phospholipids and proteins are relevant for the shorter-chained PFCAs in this study, while blood plasma proteins appear to strongly dominate partitioning for the C6 PFSA (Fig. 5).

Data from mammalian albumin studies were used to parameterize partitioning to blood plasma proteins in this model.^39^ Reasonable model performance suggests that terrestrial organism partitioning data can reasonably be extrapolated to fish blood plasma proteins. Albumin or albumin-like protein content is lower in fish compared to humans and other terrestrial organisms and comprises only approximately one-third of the volume of phospholipids in fish^55^ (ESI Section 7†). The impact of uncertainty in the blood plasma protein partitioning parameters on modeled whole-body tissue concentrations is therefore limited in magnitude by the small volume contribution of blood plasma proteins as a tissue medium (0.3%). This is particularly true for PFAAs that are strongly partitioned to phospholipids (*i.e.*, C8 PFSA, C9–C11 PFCAs). For these compounds, sensitivity ratios for both the blood plasma protein partitioning and volume parameters are low (|SR| < 0.3; ESI Section 9†). Sensitivity ratios for these parameters for PFAAs with *η*_pfc_ < 8 are higher (C6 PFSA > 0.6, C8 PFCA > 0.45), reflecting the larger contribution of blood plasma proteins to total tissue partitioning for these compounds. This suggests that uncertainty in blood plasma protein partitioning parameters could play a role in the modeled errors for the C6 PFSA and C8 PFCA but has limited impact on the other modeled PFAAs. Albumin protein binding studies show a curvilinear relationship between binding strength and PFCA chain length, potentially further decreasing the role of blood plasma protein binding for the long-chained PFCAs.^39,54,56,57^

Data on phospholipid partition coefficients and volume content are more available than for albumin protein parameters, and less variable across both studies and species^16,37,38^ (ESI Section 3†). The most bioaccumulative PFAAs (C8 PFSA, C10–C11 PFCAs) are more strongly influenced by phospholipid partitioning, with higher sensitivity ratios for the phospholipid partitioning coefficient (>0.4) compared to PFAAs with *η*_pfc_ < 8 (<0.3). Together, this suggests that current model parameterization of tissue partitioning is most robust for these more-bioaccumulative PFAAs. This contributes to better model performance for the C8 PFSA and C10–C11 PFCAs across model evaluation datasets, when compared to PFAAs with *η*_pfc_ < 8.

### Dietary consumption is a key uptake pathway across PFAAs

Model results suggest dietary consumption rather than direct PFAA uptake from water is the dominant PFAA uptake pathway for all fish in the Gironde Estuary. Dietary uptake accounted for >95% of total uptake for benthic fish (sole, flounder) and >80% of total uptake for species in the benthopelagic food web (goby, anchovy, sprat, common seabass). These results hold for all PFAAs, including those with fewer than eight perfluorinated carbons.

Strong dietary uptake of neutral POPs is typically associated with strong hydrophobicity and bioaccumulation potential (log *K*_ow_ > 5).^15^ In contrast, the dominance of modeled dietary uptake in this case study is driven primarily by the high efficiency of gut chemical absorption efficiency (>10^−1^) relative to gill chemical absorption efficiency (10^−4^ to >10^−1^). Ionizable chemicals like PFAA exhibit slow gill membrane transport rates^58^ compared to neutral POPs (gill chemical absorption efficiency >10^−1^).^46^ Aqueous uptake rates of the least hydrophobic PFAAs are orders of magnitude lower than that of neutral POPs. In contrast, dietary uptake rates for all measured PFAAs are within the same order of magnitude as neutral POPs. This is likely due to the longer residence times of PFAAs in the gut and intestines compared to the gills,^15,17,41,47^ which is further extended by enterohepatic recirculation of PFAAs.^59^ Therefore, despite the high aqueous solubility of PFAA and large volumes of water ventilated by fish, aqueous uptake for the least hydrophobic PFAAs can remain low relative to dietary uptake.

These results illuminate a mechanism that allows dietary consumption to be the dominant contributor to total chemical uptake for PFAAs, even when biomagnification is not observed.^45,46,55^ In theory, dietary absorption of contaminants will drive biomagnification in an organism that is otherwise at equilibrium with its aqueous surroundings.^60^ However, active loss pathways such as renal elimination may play a role in lowering tissue concentrations relative to expectations based on equilibrium partitioning, particularly for PFAAs with *η*_pfc_ < 8. Factors like food digestibility, organism bioenergetics, and the stochasticity of dietary uptake can influence the observation of biomagnification in field systems.^61–63^ As illustrated by the Gironde Estuary case study, these uptake patterns may be most likely in environments with elevated sediment PFAA concentrations and/or particulate loads that drive high dietary uptake at the base of the food web.

Gill membrane transport rates substantially influence the relative importance of different exposure pathways in each ecosystem, and consequently, overall model performance. The aqueous chemical absorption efficiency parameter, which describes gill membrane transport, is among the most sensitive parameters in this model, with the absolute values of the sensitivity ratios >0.6 for all PFAAs (ESI Section 9†). Mechanistic understanding of factors driving variability in gill membrane transport rates for PFAA are limited. Prior work focused primarily on the role of pH in driving diffusive transport rates.^38^ Aqueous chemical absorptions efficiencies for individual PFAAs, calculated from uptake rates measured in bioconcentration studies, vary up to two orders of magnitude among studies.^25–27,64,65^ It is unclear how much of this variability is driven by measurement uncertainty and differences in measurement methods, rather than ecosystem or organism characteristics. In addition to pH, factors such as salinity and ventilation rate have been postulated as potential sources of variability in respiratory uptake, but systematic studies of these factors have been scarce.^25,66,67^ Additional data on gill membrane transport and drivers of variability in gill uptake would improve model performance and generalizability, as well as our understanding of the relative importance of dietary exposure pathways.

### Bioconcentration of ionizable PFAA alternatives

Since the mid-2000s, PFAS production has been increasingly shifting from PFAAs toward fluoroalkyl compounds with shorter carbon chains and new PFAS chemistries.^1,2,68^ However, in some cases fluorinated alternatives that were designed to be less bioaccumulative or to degrade into short-chained PFAAs also bioaccumulate^34,69,70^ and models for these alternative compounds are needed. We evaluated model performance for an alternative compound that has been observed to bioaccumulate in aquatic organisms (9-Cl-PF3ONS), and one that has not (HFPO-DA).^34,43,44,69^

Modeled BCFs for both HFPO-DA and 9-Cl-PF3ONS fell within a factor of two of observed concentrations, without modeled metabolism or renal elimination (Tables S6a and b†). This suggests that the current parameterization of tissue partitioning and aqueous chemical absorption efficiency are sufficient to represent whole-body bioaccumulation behavior of these compounds. Prior work showed that predicted surface charge densities for these ether-linked compounds were similar to their analogous PFAAs with similar chain lengths (C4–C6 PFCAs and C8–C9 PFSAs, respectively), resulting in similar partitioning behavior as PFAAs for both phospholipids and albumin^38,39^ (ESI Section 6†). Estimated aqueous chemical absorption efficiencies were also within the range of expected values for the alternatives based on chain length alone, although directly comparable empirical measurements for the HFPO-DA are not available. Low aqueous chemical absorption efficiencies for the HFPO-DA relative to the C8 PFCA (an order of magnitude lower) may be due to its comparatively shorter-chained (C6) and branched structure. Similar patterns have been observed for the C8 PFSA, with lower uptake rates observed for the branched *versus* linear isomers.^42^

We hypothesize that the apparent lack of a role for modeled renal elimination in these PFAA alternatives is driven by their perfluorinated carbon chain structures. For 9Cl-PF3ONS, the lack of renal elimination is consistent with our results for the PFAAs with a similar or longer perfluorinated chain length. For HFPO-DA, the short fluoroalkyl-chain may also result in weak binding to renal elimination proteins. Steric hindrance from branched carbon chain tails has been observed to result in weaker albumin protein binding for branched C8 PFSA compared to the linear isomer,^71^ and a similar phenomenon could also be contributing to slightly weaker protein binding relative to patterns observed for PFCAs. HFPO-DA partitioning to albumin proteins, for example, is weaker than for the C6 PFCA.^39^ PFCAs with <6 perfluorinated carbons did not appreciably bind to rat renal elimination proteins in *in vitro* protein transport studies, suggesting the same could be true for HFPO-DA.^22,39^ Overall, model results suggest that the low bioaccumulation of HFPO-DA can be explained by low sorption to tissue matrices alone.

This example demonstrates the model's applicability to a common class of PFAA alternatives, the perfluoroalkyl ether acids. Experimental studies highlight the potential for variability in bioaccumulation patterns for new PFAS chemistries, but also demonstrate the ongoing need for empirical studies to improve our understanding of structure–activity relationships driving partitioning and membrane transport processes. Studies linking experimental data and molecular models across a wider range of functional group substitutions can play an important role in building our understanding of structure–activity relationships that will ultimately improve our ability to predict key bioaccumulation behaviors for alternative PFAS classes.

### Implications for future work

The model developed in this study currently performs best for the most bioaccumulative PFAAs and their alternatives (C8 PFSA, C10–C11 PFCAs, 9-Cl-PF3ONS). For these compounds, our analysis suggests that renal elimination plays a limited role, and therefore whole-body concentrations can be reasonably described by equilibrium partitioning processes. Modeled median concentrations fell within a factor of three of observed concentrations across whole-body organism concentrations spanning 2–3 orders of magnitude. While PFAS production has increasingly shifted towards shorter-chained compounds,^1,2,68^ field studies show that these more bioaccumulative compounds continue to dominate measurable PFAS body burdens, due to their extensive historical use, environmental persistence, and comparatively greater bioaccumulative potential.^72,73^

Based on the performance indicators discussed in this study, the model developed here can assist in understanding patterns of accumulation of these more-bioaccumulative PFAS in fish within and across ecosystems. It is relatively simple, enabling adaptation for regulatory and risk assessment contexts, including screening-level risk assessments and the development of environmental quality guidelines.^74^ For example, fish consumption advisories have been developed in several states, thus far primarily for the C8 PFSA.^9,10,13,75–77^ In states where health-based guidelines have been developed for multiple PFAS, the C8 PFSA remains the most commonly detected PFAS that drives the development of advisories.^10,13^ This model can be used as a tool to predict tissue C8 PFSA concentrations in diverse ecosystems. It can also identify systems at higher risk for PFAA bioaccumulation that can be prioritized for further monitoring or risk assessment.

Bioaccumulation models for shorter-chained PFAAs and PFAA alternatives are increasingly needed. Our analysis highlights key opportunities to further improve mechanistic understanding of structure–activity relationships that can inform a more generalizable estimation of key model parameters. Overall, our results suggest that both fish blood plasma protein and renal elimination protein binding are important bioaccumulation drivers for shorter-chained compounds, including the C8 PFCA and C6 PFSA. Renal elimination in fish may be of limited importance for the shortest-chained (<6 perfluorinated carbons) compounds due to weak binding to renal elimination proteins. Additional characterization of renal elimination rates and associated protein transport mechanisms are needed to further evaluate these hypotheses. More generally, additional empirical measurement of protein binding mechanisms is a priority for further model development, given the sensitivity of these mechanisms to functional group variations in newer PFAS classes, and the potential for non-linear chain-length binding patterns.^18,19,78^ Continued development of computational models for predicting these parameters across PFAS groups will also play an important role in facilitating the extension of this model to a growing number of shorter-chained PFAS.

Improved estimates of aqueous and dietary absorption efficiencies for PFAS based on cellular uptake or membrane permeability studies are also greatly needed. These parameters are among the most sensitive model parameters for all PFAS, but are highly variable and poorly characterized. The empirically-based parameter values used in this study deviate from the pH- and *K*_ow_-dependent patterns presented in previous models of membrane transport for ionizable or neutral chemicals,^15,16^ but sparse PFAA-specific data currently limits the development of a mechanistic parameter sub-model that accounts for potential drivers of variability, such as salinity, pH, or active transport. Characterization of these key membrane transport rates across PFAS structures, as well as potential environmental and organism characteristics driving variability in these parameters, are needed to both improve model adaptability across a range of environmental conditions, and aid in model extension to emerging PFAS classes.

## Conflicts of interest

There are no conflicts to declare.

## Supplementary Material

EM-024-D2EM00047D-s001
