# Supplementary material for: A food web bioaccumulation model for the accumulation of per- and polyfluoroalkyl substances (PFAS) in fish: how important is renal elimination?
Source: Environ Sci Process Impacts. 2022 May 25;24(8):1152–64. doi: 10.1039/d2em00047d (PMC9384792; doi:10.1039/d2em00047d)
Supplement: EM-024-D2EM00047D-s001 [file EM-024-D2EM00047D-s001.pdf]

Electronic Supplementary Material (ESI) for Environmental Science: Processes & Impacts.
This journal is © The Royal Society of Chemistry 2022

A food web bioaccumulation model for the accumulation of poly- and perfluoroalkyl substances (PFAS) in fish: How important is renal elimination?

Supplementary Information

Section 1. General model description

Model code can be accessed at: https://github.com/SunderlandLab/fish_foodweb_pfas_model

Table S1. Model equations

Parameter	Description	Equation or Value	Units	Ref
$C_{b,\,fish}$	Contaminant concentration in the whole body for fish and invertebrates	$\frac{k_1 C_W + k_D C_D}{k_2 + k_E + k_G + k_M}$	ng/g	1
$C_{b,\,phyto}$	Contaminant concentration in phytoplankton	$\frac{k_1 C_{WTO} \emptyset}{k_2 + k_G + k_M}$	ng/g	1
Environmental exposure				
C_W	Contaminant concentration in water (weighted average between overlying and porewater)	$m_0 \emptyset C_{WTO} + (1 - m_0) C_{WDP}$	ng/mL	1
C_{WTO}	Chemical concentration in overlying water	Model input, varies by ecosystem	ng/mL	-
C_{WDP}	Chemical concentration (dissolved) in sediment porewater	$\frac{C_S}{OCS} \div K_{oc}$	ng/mL	1
m_0	Fraction of organism respiration from overlying water	Varies by organism, see Tables S4a and S5a	unitless	-
$\emptyset$	Chemical fraction in the dissolved phase	Varies by ecosystem, see Tables S4b and S5b	unitless	varies
OCS	Organic carbon content in sediment	Varies by ecosystem, see Table S5b	kg/L	varies
D_{oc}	Organic carbon – water partitioning coefficient	Varies by ecosystem, see Table S2	L/kg	varies
C_D	Contaminant concentration in the diet (weighted average between prey items and sediment)	$\Sigma P_i C_{D,i} + P_d C_s$	ng/g	1
C_S	Contaminant concentration in sediment	Model input, varies by ecosystem	ng/g	-
P_i	Proportion of diet composed of prey item, i	Varies by food web, see Tables S5c	unitless	-
P_d	Proportion of diet composed of sediment	Varies by food web, see Tables S5c	unitless	-
Tissue fractions[b]				

υ_{NG}	Neutral lipid fraction in the gut	$\frac{(1-\varepsilon_N)\upsilon_{ND}}{\{(1-\varepsilon_N)\upsilon_{ND}+(1-\varepsilon_L)\upsilon_{LD}+(1-\varepsilon_P)\upsilon_{PD}+(1-\varepsilon_O)\upsilon_{OD}+(1-\varepsilon_W)\upsilon_{WD}\}}$	--	1,a
υ_{LG}	Phospholipid fraction in the gut	$\frac{(1-\varepsilon_L)\upsilon_{LD}}{\{(1-\varepsilon_N)\upsilon_{ND}+(1-\varepsilon_L)\upsilon_{LD}+(1-\varepsilon_P)\upsilon_{PD}+(1-\varepsilon_O)\upsilon_{OD}+(1-\varepsilon_W)\upsilon_{WD}\}}$	--	1,a
υ_{PG}	Binding protein fraction in the gut	$\frac{(1-\varepsilon_P)\upsilon_{PD}}{\{(1-\varepsilon_N)\upsilon_{ND}+(1-\varepsilon_L)\upsilon_{LD}+(1-\varepsilon_P)\upsilon_{PD}+(1-\varepsilon_O)\upsilon_{OD}+(1-\varepsilon_W)\upsilon_{WD}\}}$	--	1,a
υ_{OG}	NLOM fraction in the gut	$\frac{(1-\varepsilon_O)\upsilon_{OD}}{\{(1-\varepsilon_N)\upsilon_{ND}+(1-\varepsilon_L)\upsilon_{LD}+(1-\varepsilon_P)\upsilon_{PD}+(1-\varepsilon_O)\upsilon_{OD}+(1-\varepsilon_W)\upsilon_{WD}\}}$	--	1,a
υ_{WG}	Water fraction in the gut	$\frac{(1-\varepsilon_W)\upsilon_{WD}}{\{(1-\varepsilon_N)\upsilon_{ND}+(1-\varepsilon_L)\upsilon_{LD}+(1-\varepsilon_P)\upsilon_{PD}+(1-\varepsilon_O)\upsilon_{OD}+(1-\varepsilon_W)\upsilon_{WD}\}}$	--	1,a
υ_{ND}	Neutral lipid fraction in the diet	$\Sigma P_i \upsilon_{NB,i}$	--	1
υ_{LD}	Phospholipid fraction in the diet	$\Sigma P_i \upsilon_{LB,i}$	--	1
υ_{PD}	Binding protein fraction in the diet	$\Sigma P_i \upsilon_{PB,i}$	--	1
υ_{OD}	NLOM fraction in the diet	$\Sigma P_i \upsilon_{OB,i}$	--	1
υ_{WD}	Water fraction in the diet	$\Sigma P_i \upsilon_{WB,i}$	--	1
Tissue partitioning				
X_N	Neutral fraction of the compound	$(1+10^{pH_i - pKa})^{-1}$	--	2
X_I	Ionic fraction of the compound	$1 - X_N$	--	2
$\log K_{OW,I}$	Octanol-water partitioning coefficient for the ionic chemical form	$\log K_{OW,N} - 3.1$	L/kg	2
D_{OW}	Octanol-water distribution coefficient	$X_N K_{ow,N} + X_I K_{ow,I}$	L/kg	2
β	Organic carbon (phytoplankton) or NLOM proportionality constant	0.35 (phytoplankton)<br>0.05 (all others)	--	1,2
D_{GW}	Gut-water distribution coefficient[b]	$\upsilon_{NG}D_{OW} + \upsilon_{LG}D_{MW} + \upsilon_{PG}K_{PW} + \beta\,\upsilon_{OG}D_{ow} + \upsilon_{WG}$	L/kg	1, a
D_{BW}	Body-water distribution coefficient[b]	$\upsilon_{NB}D_{OW} + \upsilon_{LB}D_{MW} + \upsilon_{PB}D_{PW} + \beta\,\upsilon_{OB}D_{ow} + \upsilon_{WB}$	L/kg	1, a

D_{GB}	Gut-body partitioning coefficient	D_{GW} / D_{BW}	kg/kg	1
Respiration				
$k_{1,phyto}$	Uptake rate constant for phytoplankton	$(A + \frac{B}{D_{MW}})^{-1}$	L/kg/d	1,a
A	Resistance to chemical uptake through the aqueous phase of phytoplankton	6 x 10^{-5}	d	1
B	Resistance to chemical uptake through the organic phase of phytoplankton	5.5	d	1
$k_{1,fish}$	Respiratory (i.e. gill) uptake rate constant for invertebrates and fish	$E_W G_V / W_B$	L/kg/d	1
E_W	Chemical absorption efficiency across the gill membrane (aqueous chemical absorption efficiency)	$k_{1,empirical}\, W_B / G_{V,}$ where $k_{1,empirical}$ is from ref. 3	unitless	3,4
G_V	Ventilation rate	$1400 {W_B}^{0.65} / C_{OX}$	L/d	1
k_2	Respiratory elimination rate constant	k_1 / D_{BW}	d^{-1}	1
Ingestion / Egestion				
k_D	Dietary uptake rate constant	$E_D G_D / W_B$	kg/kg/d	1
E_D	Chemical absorption efficiency across the gut membrane (gut or dietary chemical absorption efficiency)	Empirically calculated, see Table 2a	unitless	5,6
$G_{D,filter}$	Feeding rate for filter feeders (invertebrates)	$G_V C_{SS} \sigma$	kg/d	1
$G_{D,fish}$	Feeding rate for coldwater fish	$0.022\, {W_B}^{0.85} e^{0.06T}$	kg/d	1
σ	Scavenging efficiency of particles absorbed from the water	1	unitless	1
k_E	Fecal elimination rate constant	$G_F E_D D_{GB} / W_B$	d^{-1}	1
G_F	Fecal egestion rate	$\{(1-\varepsilon_N)\upsilon_{ND} + (1-\varepsilon_L)\upsilon_{LD} + (1-\varepsilon_P)\upsilon_{PD} + (1-\varepsilon_O)\upsilon_{OD} +$	kg/d	1,a
k_R	Renal elimination rate constant	Calculated, see Section 7	d^{-1}	a
Other organism parameters				
$k_{G,\,phyto}$	Growth rate constant for phytoplankton	GRF	d^{-1}	1
$k_{G,\,fish}$	Growth rate constant for invertebrates and	$GRF \cdot {W_B}^{-0.2}$	d^{-1}	1

	fish			
GRF	Growth rate factor	Constant, see Tables S4a and S5a	varies	-
k_M	Metabolic elimination rate through biotransformation	0	d^{-1}	-
pH_i	Organism internal pH	7.4	unitless	2

a – This study

b – See Sections 4 and 5 for individual parameter values

Section 2. PFAA parameters

Table S2. PFAA parameters

	**C6 PFSA**	**C8 PFSA**	**C8 PFCA**	**C9 PFCA**	**C10 PFCA**	**C11 PFCA**	**Reference**
pKa	0	0	1	1	1	1	2
Partitioning Coefficients							
$\log K_{OW,N}$	5.20	6.43	5.30	5.92	6.50	7.15	COSMOtherm 2011, following ref 2 [a]
$\log D_{OW}$	2.10	3.33	2.20	2.82	3.40	4.05	Calculated from $K_{OW,N}$, following ref 2
$\log D_{MW}$	3.82	4.88	3.51	4.04	4.63	*5.22* [b]	7
$\log D_{PW}$	4.94	4.81	4.33	4.46	4.86	4.74	Allendorf et al. 2019[c]
$\log D_{OC,\ estuarine}$[d]	3.70	4.34	4.05	4.38	4.58	4.99	Munoz et al. 2017
Chemical transfer efficiencies							
$E_{W,\ empirical}$	7.9×10^{-4}	6.8×10^{-2}	6.8×10^{-4}	*4.9×10^{-3}*	3.7×10^{-2}	1.53×10^{-1}	3[e]
$E_{D,juv}$	0.7	1	0.59	1	1	1	5
$E_{D,\ adult}$	0.558	0.721	0.138	0.522	*0.65*	*0.75*	6[f]

a – Dry basis, see Table S5a $\log K_{OW,N}$

b – Empirical data is not available for PFUA. Droge found consistent increases in D_{MW} of log unit 0.59 increments for each additional fluorinated carbon, which was used to estimate a value for D_{MW} for PFUA. For further discussion of D_{MW} parameter selection, see Table S3a.

c – Partitioning coefficient assumes a protein density of 1.36 kg/L to convert from units of kg/L to L/L.

d – Empirical values from the same or similar study site should be used whenever possible.

e – Values were calculated based on calculation from reported uptake rate, assuming a fish weight of 7g and oxygen concentration of 7.23 mg/L. The italicized value was estimated from an uptake rate that was estimated from a log-linear regression between chain length and uptake rate.

f– Italicized values are extrapolated based on approximate chain-length patterns. In the current study, $E_{D,juv}$ is used to parameterize the BCF and BMF model applications, while $E_{D,\ adult}$ is used to parameterize the food web model application.

Section 3. Selection of key PFAA parameters

Revisions to the partitioning and chemical transfer efficiency parameters in the Armitage et al.[2] fish bioconcentration model, made to improve applicability to food web bioaccumulation of PFAAs, are discussed below. In Tables S3a-b, rows highlighted in grey represent parameter values used in the current model.

<u>Tissue partitioning</u>

In this model, the body-water partitioning coefficient (D_{BW}) parameter used by Armitage et al.[2] was updated (1) to revise the parameterization of phospholipid partitioning and (2) to include partitioning to blood plasma binding proteins.

Phospholipids -- Partitioning to phospholipids is described by the membrane-water partitioning coefficient D_{MW}. In this model, $D_{MW}$is parameterized using empirical values from a laboratory-based partitioning study using solid-supported phosphatidylcholine lipid bilayer membranes designed to mimic intestinal epithelium[7]. Similar D_{MW} values were measured in a second laboratory study for all PFAAs except the C11 PFCA, for which the measured D_{MW} was lower than the C10 PFCA[4]. However, the second study was inconclusive as to whether this was due to the lower diffusibility of larger compounds or experimental error, and therefore this experimental value is not used in the current study. Empirically measured Dmw values are also similar to those calculated by the mechanism proposed in the ionogenic model, when based on Kow estimates from COSMOtherm (2011), suggesting that these modeled D_{MW} values may be reasonable approximations for compounds without empirical Dmw measurements (see Table S3a).

Following Armitage et al.[2], the volume fraction of phospholipids (υ_{LB}) is estimated at 1% in fish. In this study, υ_{LB} is approximated at 1% in all other organisms as well, although it is likely that υ_{LB} may be more variable in lower trophic level organisms, particularly phytoplankton.

Table S3a. Log Dmw

Data Source	C6 PFSA	C8 PFSA	C8 PFCA	C9 PFCA	C10 PFCA	C11 PFCA
Empirical						
**Droge 2019[7]**	**3.82**	**4.88**	**3.51**	**4.04**	**4.63**	***5.22[a]***
Ebert et al. 2020[4]	4.13	4.89	3.52	4.25	4.82	4.54[b]
Calculated from Kow following Armitage et al. 2013[2]						
COSMOtherm 2011	3.37	4.61	3.47	4.10	4.69	5.34
KowWIN 1.68	1.31	2.65	2.98	3.65	4.33	5.01
Arp, Niedler & Goss	--	3.47	1.76	2.67	3.57	4.58

a – Value is extrapolated based on chain-length pattern for PFCAs
b – Value does not follow chain-length patterns.

Albumin proteins - PFAA binding to albumin is widely believed to drive high concentrations observed in blood plasma, and has been measured with human and bovine serum albumin. Albumin binding patterns show significant species-specific variation[8], but PFAA partitioning coefficients have not been measured for any fish-specific proteins. Available binding and partitioning studies using bovine serum albumin (BSA) and human serum albumin (HSA) have shown widely variable results due to a diversity of measurement methods, including poor standardization of ligand (i.e. chemical) and receptor (i.e. protein) concentrations used during measurement[8]. The three available albumin partitioning studies show contrasting patterns for headgroup and PFCA chain length partitioning to BSA. Bischel et al.[9] reported decreasing partitioning strength with increasing chain length for the C8-C12 PFCAs, and nearly identical partitioning coefficients for identical chain length PFCAs and PFSAs, whereas Allendorf et al.[10] and Aleiso et al.[11] found increasing partitioning strength with increasing chain length, and greater chain-length dependence for PFCAs compared to PFSAs (Table S3b). Allendorf et al.[10] attributed discrepancies to differences in study design: all studies used equilibrium dialysis, but Bischel et al.[9] conducted their experiment at a higher PFAA:albumin molar ratios, such that over 98% of PFAAs were bound at equilibrium, which can in turn lead to oversaturation of protein binding sites that leads to an underestimation of true

partitioning strength. The pattern observed by Allendorf et al.[10] also align more closely with patterns of fish blood protein binding strength measured by Zhong et al.[12] in carp, as well as overall patterns of bioconcentration and biomagnification observed in laboratory studies of fish[3,5], in which accumulation or partitioning is stronger for PFSAs compared to PFCAs of the same chain length. We use values from Allendorf et al.[10] in this model.

The presence and abundance of albumin proteins in fish can be highly variable, with albumin making up anywhere from 0 to 60% of total blood proteins in different species[13,14]. The volume fraction of albumin binding proteins is estimated to range between 0 to 0.4% in fish (SI Section 7) based on literature values for albumin or albumin-like protein concentrations and tissue volumes estimated for an average fish. In this model, the volume fraction of albumin or albumin-like binding proteins is set at 0.3% for fish, 0.1% for invertebrates, and 0% in phytoplankton.

Table S3b. Log Dpw

Data Source	C6 PFSA	C8 PFSA	C8 PFCA	C9 PFCA	C10 PFCA	C11 PFCA
**Allendorf et al. 2020[a]**	**4.94**	**4.81**	**4.33**	**4.46**	**4.86**	**4.74**
Bischel et al. 2011	4.3	4.1	4.14	4.05	3.86	3.7
Alesio et al. 2022	5.05	5.53	4.82	5.93	6.10	--

a – Partitioning coefficient assumes a protein density of 1.36 kg/L to convert from units of kg/L to L/L.

Liver fatty acid binding proteins - PFAAs have also been shown to bind to liver- and other fatty-acid binding proteins, which are considered to be drivers of elevated PFAA concentrations in the liver and kidney. The pattern of human L-FABP binding strength across PFAA structures is similar to the pattern observed for fish blood proteins, with higher binding affinities for PFSAs compared to PFCAs of the same chain length, and increasing binding affinities with chain length for the C4 to C8 PFSAs and C7 to C11 PFCAs[12,15]. No partitioning coefficients have been measured for any L-FABP, and no published studies have measured PFAA binding to L-FABP in fish species, but lower binding strengths have been reported for human L-FABP compared to albumin[8,11,15,16]. The relative abundance of L-FABP is also substantially lower than that of albumin[17]. We estimate that the contribution of L-FABP to total PFAA binding is not larger than uncertainties in albumin binding strength and abundance (Section 7). Therefore, L-FABP and other potential binding proteins are not explicitly included in this model.

Neutral storage lipids and NLOM -- Binding to neutral storage lipids and NLOM is described following Armitage et al.[2], using the octanol-water distribution coefficient (D_{OW}). D_{OW} in the default model is calculated from Kow estimated with COSMOtherm (2011).

<u>Chemical absorption efficiencies</u>

Branchial uptake and elimination – Membrane transport of ionized compounds is generally significantly reduced compared to that of neutral POPs, with transport dominated by the neutral fraction of the compound. For weakly ionized compounds, diffusive transport by the neutral chemical fraction results in pH-dependent transport rates. However, for highly ionized compounds, the relative importance of other transport mechanisms, such as paracellular transport, protein-mediated or ion channel transport, or mass transfer of the ionic fraction, are hypothesized to increase as the size of the neutral chemical fraction grows negligibly small. Saarikoski et al.[18] found that for highly ionized compounds, as pH increases chemical uptake initially decreases but then plateaus**.** Armitage et al.[2] updated the Arnot & Gobas[1] submodel for aqueous chemical transfer efficiency (E_W) for ionic chemicals to reflect these behaviors by (1) modeling the pH-dependence of diffusive transport and (2) adding a constant to crudely account for non-diffusive transport pathways, which increase in importance as diffusive transport rates decrease for highly ionized compounds.

In a series of membrane permeability experiments using phosphatidylcholine liposomes, Ebert et al.[4] found *in vitro* lipid bilayer membrane permeabilities to be consistent with pH-independent passive transport for PFSAs, and only weakly pH-dependent passive transport for PFCAs. For PFSAs, the effective permeability of the ionic fraction is about eight orders of magnitude greater than that of the neutral fraction. The empirically measured permeabilities matched permeabilities calculated from cellular uptake studies quite well, suggesting they reasonably represented real behaviors in cells[4,17]. Model-predicted permeabilities using either correlation with the hexadecane-water partitioning coefficient or COSMOtherm similarly suggest that permeability of the ionic fraction of PFSA is orders of magnitude greater than the effective neutral permeability at biological pH. The authors attributed greater permeability of the ionic fraction of PFSAs compared to PFCAs (SI Tables 3.2.1, 3.2.7; Figures 3.2.2-3) [4] to the broader surface charge distribution in the sulfonate compared to the carbonate headgroup, resulting in lower surface charge densities and lower resistance to transport through the neural interior of the bilayer membrane. This indicates pH-independent membrane transport, as PFSAs are predominantly in their ionic form at environmental pH.

For PFCAs, both the neutral and ionic chemical fractions play a role in transport, with up to an order of magnitude difference between the effective membrane permeability for the neutral and ionic fractions. This suggests that pH-dependent transport of the neutral chemical fraction may play a larger role for PFCAs, but at much lower rates than expected based on models developed for more weakly ionized compounds[2]. While the Armitage et al.[2] model predicts a two order of magnitude difference in permeability for PFCAs between pH 6 and 8, Ebert et al. [4] estimated a variability of only about 30%, based on passive uptake rates measured *in vitro*

in human HEK293 cells[19]. Here we approximate membrane transport of all PFAAs with a pH-independent parameterization of membrane transport at environmentally relevant pH.

The E_W parameter is estimated from branchial uptake rates from lab bioconcentration studies based on the modeled relationship between these values and gill ventilation rate (see Table S1). Ew values calculated from the Martin et al.[3] bioconcentration study were based on reported uptake rates and gill ventilation rates calculated based on a reported average fish weight of 7.3g and an estimated oxygen concentration of 10 mg/L (92% dissolved oxygen saturation at 12 °C). The uptake rate for the C9 PFCA was not directly measured, and was therefore instead interpolated from a linear regression between uptake rate and PFCA chain length. For the current model, E_W calculated from the laboratory bioconcentration study is directly used in all model applications based on an assumption that aqueous chemical transfer efficiency does not vary substantially with pH and therefore is comparable between all study conditions. In general, the empirically estimated $E_W$values follow membrane permeability patterns observed in other literature, showing that total permeability is greater for PFSAs than PFCAs, and that permeability increases with chain length (studies cited in Ng & Hungerbuhler[17], Ebert et al.[4]).

Gut uptake and elimination – Gut membrane chemical transfer efficiencies (E_D, also referred to as absorption or chemical assimilation efficiencies) are generally not observed to be significantly different for ionogenic chemicals compared to neutral chemicals, due to the long residence time of chemicals in the gut that enables a longer period for transport to occur compared to gill membrane transport[20]. However, the submodel for dietary absorption efficiencies in the bioaccumulation model for neutral organic chemicals is based on an inverse relationship between absorption efficiency and hydrophobicity[1], whereas absorption efficiency values modeled from uptake rates measured in laboratory biomagnification studies on both adult- and juvenile rainbow trout show dietary absorption efficiency increases with hydrophobicity[5,6]. Thus, empirical values from these laboratory studies are used in this current study.

E_D for juvenile rainbow trout was estimated by the experimental study authors by fitting growth-corrected fish concentration data to a kinetic rate equation for constant dietary exposure. Reported values therefore may reflect potential measurement errors for fish and food concentrations, experimental and model assumption errors, and statistical artefacts of averaging across natural variability in experimental values. This may include factors like the assumption of a steady feeding rate, the assumption that 90% of steady state was reached during the experiment, and growth rate-correction for fish concentrations. Reported assimilation efficiencies were greater than 100% (i.e. E_D > 1) for the C8 PFSA and C10-13 PFCAs, which is not physically possible. In this study, E_D values were set at 1 when assimilation efficiencies were greater than 100%. Although these E_D values likely contain some error, we use them as reported in our biomagnification model, because our model relies on many of the same inputs and assumptions as the model used to estimate E_D (i.e. fish concentrations, food concentrations, feeding rate). By using these

parameters together, we may correct for co-occuring errors, while still accurately reflecting measured biomagnification and depuration rates that we then attribute to specific tissue partitioning and elimination mechanisms, respectively.

Other studies corroborate the overall finding that dietary assimilation efficiencies for PFAAs in fish are high. Assimilation efficiencies for adult trout more physically plausible, but still high (> 0.1) compared to branchial assimilation efficiencies. These values are used in the current study for the model application to field food web data. High dietary assimilation efficiencies compared even to neutral POPs (i.e. > 0.5) have also been assumed in other toxicokinetic models of PFAS uptake, such as in humans[21,22]. In each of these studies, high assimilation efficiencies have been attributed to enterohepatic recirculation.

Section 4. Parameterization of the controlled laboratory study model applications for PFAAs

Table S4a. Biotic state variables

Parameter	Description	Value
W_B	Weight (kg)	7.3×10^{-3} (BCF)<br>2.54×10^{-3} (BMF)
m_o	Fraction of respiration from overlying water	1
υ_{NB}	Neutral lipid fraction in the gut	0.04
υ_{LB}	Phospholipid fraction in the gut	0.01
υ_{PB}	Binding protein fraction in the gut	0.003
υ_{OB}	NLOM fraction in the gut	0.15
υ_{WB}	Water fraction in the gut	0.797
ε_N	Neutral lipid absorption efficiency	0.92
ε_L	Phospholipid absorption efficiency	0.92
ε_P	Protein absorption efficiency	0.92
ε_O	NLOM absorption efficiency	0.6
ε_W	Water absorption efficiency	0.7
GRF	Growth rate factor	--[a]

a – Growth rate was already accounted for in the reported uptake values. Therefore, growth dilution was not included as an elimination pathway in these models.

Table S4b. Environmental state variables

Parameter	Description	Value	Units
C_{ox}	Dissolved oxygen concentration	10	mg/L
T	Temperature	12	°C
pH	pH	7	
ϕ	Fraction of chemical in the dissolved phase	1[a]	

a – Particulate organic matter in the laboratory study was assumed to be negligible, and therefore 100% of the measured exposure concentration in water was estimated to be in the dissolved phase.

Table S4c. Diet composition

Parameter	Description	Values
υ_{ND}	Neutral lipid fraction in the gut	0.012
υ_{LD}	Phospholipid fraction in the gut	0.003
υ_{PD}	Binding protein fraction in the gut	0
υ_{OD}	NLOM fraction in the gut	0.15
υ_{WD}	Water fraction in the gut	0.835

1 – Following Armitage et al. 2013[2]

Section 5. Parameterization of the Gironde Estuary field study model application

Table S5a. Biotic state variables

	Phyto-plankton	Invertebrates						Fish					
	**Phy**	**Cop**	**Mys**	**Pfs**	**Gam**	**Rag**	**WSh**	**Gob**	**Acy**	**Spr**	**Sol**	**Fln**	**CSb**
W_B **(kg)**		1x10^{-7}	1x10^{-6}	1x10^{-2}	1x10^{-5}	1x10^{-5}	1x10^{-5}	5x10^{-2}	5x10^{-2}	5x10^{-2}	0.15	0.15	0.2
m_o	1	1	0.95	0.5	1	1	1	1	1	1	1	1	1
υ_{NB}	0.02	0.02	0.02	0.02	0.02	0.02	0.02	0.04	0.04	0.04	0.04	0.04	0.04
υ_{LB}	0.01	0.01	0.01	0.01	0.01	0.01	0.01	0.01	0.01	0.01	0.01	0.01	0.01
υ_{PB}	0	0.001	0.001	0.001	0.001	0.001	0.001	0.003	0.003	0.003	0.003	0.003	0.003
υ_{OB}[1]	0.15	0.15	0.15	0.15	0.15	0.15	0.15	0.15	0.15	0.15	0.15	0.15	0.15
υ_{WB}	0.82	0.819	0.819	0.819	0.819	0.819	0.819	0.797	0.797	0.797	0.797	0.797	0.797
ε_N		0.75	0.75	0.75	0.75	0.75	0.75	0.92	0.92	0.92	0.92	0.92	0.92
ε_L		0.75	0.75	0.75	0.75	0.75	0.75	0.92	0.92	0.92	0.92	0.92	0.92
ε_P		0.75	0.75	0.75	0.75	0.75	0.75	0.92	0.92	0.92	0.92	0.92	0.92
ε_O		0.6	0.6	0.6	0.6	0.6	0.6	0.6	0.6	0.6	0.6	0.6	0.6
ε_W		0.55	0.55	0.55	0.55	0.55	0.55	0.55	0.55	0.55	0.55	0.55	0.55
**GRF**	8x10^{-2}	3.5x10^{-4}	3.5x10^{-4}	3.5x10^{-4}	3.5x10^{-4}	3.5x10^{-4}	3.5x10^{-4}	1.4x10^{-3}	1.4x10^{-3}	1.4x10^{-3}	1.4x10^{-3}	1.4x10^{-3}	1.4x10^{-3}

1 – This parameter represents organic carbon for phytoplankton and NLOM for all other species. Partitioning to these different fractions utilizes different proportionality constants β (see Table S1). The NLOM proportionality constant is estimated to be between 0.025 and 0.05[2], while the OC proportionality constant is estimated to be 0.35[1].

Table S5b. Environmental state variables

Parameter	Description	Value		Units
C_{ox}	Dissolved oxygen concentration	7.5		mg/L
T	Temperature	12		°C
pH	pH	8		unitless
OCS	Organic carbon content in sediment	0.0215		kg/L
ϕ	Fraction of chemical in the dissolved phase[a]	C6 PFSA	0.92	unitless
		C8 PFSA	0.4	
		C8 PFCA	0.8	
		C9 PFCA	0.6	
		C10 PFCA	0.35	
		C11 PFCA	0.25	

a – Fraction in the dissolved phase based on empirical values reported in the Gironde Estuary system (see Figure 3)[23].

Table S5c. Diet Table, Gironde Estuary

	Sed	Phy	Cop	Mys	Pfs	Gam	Rag	WSh	Gob	Acy	Spr	Sol	Fln	CSb
**Phy**	0													
**Cop**	0	1												
**Mys**	0.1	0.45	0.45											
**Pfs**	0.3	0.65	0.05	0										
**Gam**	0.3	0.35	0.35	0	0									
**Rag**	0.9	0.05	0.05	0	0	0								
**WSh**	0	0.3	0.3	0.4	0	0	0							
**Gob**	0	0.01	0.09	0.52	0	0.18	0.09	0.11						
**Acy**	0	0	0.8	0.2	0	0	0	0	0					
**Spr**	0	0.05	0.995	0	0	0	0	0	0	0				
**Sol**	0	0	0	0	0	0.01	0.95	0.04	0	0	0			
**Fln**	0	0.1	0	0.001	0	0.06	0.903	0.026	0	0	0	0		
**CSb**	0	0	0.003	0.007	0	0.32	0.01	0.472	0	0.047	0	0.141	0	

Section 6. PFAA alternatives BCF model application – parameterization and model results

Dietary uptake and elimination were not included in this calculation. Feed was not spiked with the contaminant, so dietary uptake should be negligible, affected only by partitioning into the feed from aqueous solution. Fecal elimination is expected to be minimal, based on results from the bioconcentration model for PFAAs (<1% of total elimination). Tissue composition and absorption efficiencies are the same as values in the PFAA model applications (Table S4a).

Table S6a. 9Cl-PF3ONS BCF model parameterization & results

Parameter	Value	Units	References or Source
**Chemical parameters**			
Log Dmw	5.14		[24]
Log Dpw	5.14		[24]
Log Kow	5.24		[25] (KowWIN 1.69)
Log Dow	2.14		Calculated following Ref 2
Log Dbw	3.25		Calculated, Table S1
Ew	0.015		Approximated from k_1 in Ref [26]
**Organism & Environmental parameters**			
Wb	0.0004	kg	[27,28; a]
Cox	7.44	mg/L	[b]
T	25	C	[26]
**Calculated rates**			
Gv	1.16	L/d	Calculated (see Table S1)
k1	43.6	L/kg/d	Calculated (see Table S1)
k2	2.42×10^{-2}	d^{-1}	Calculated (see Table S1)
kg	2.5×10^{-2}	d^{-1}	Estimated from Figure 4[26]
**Bioconcentration factor**			
Log BCF (Model)	2.95	L/kg	k1 / (k2 + kg)
Log BCF (Obs)	3.06	L/kg	[26]
**Model Bias**	**0.77**		

a – Weight is not reported. Value estimated from other studies of 2-month-old Chinese rare minnow.
b – Oxygen content was not reported. Assumed 90% oxygen saturation.

Table S6b. HFPO-DA BCF model parameterization & results

Parameter	Value	Units	References or Source
**Chemical parameters**			
Log Dmw	2.41		[24]
Log Dpw	3.19		[24]
Log Kow	3.36		[25] (KowWIN v1.69)
Log Dow	0.26		Calculated following Ref 2
Log Dbw	0.86		Calculated, Table S1
Ew	6×10^{-5}		Approximated from k_1[a] in Ref [29]
**Organism & Environmental parameters**			
Wb	0.005	kg	[29]
Cox	8.3	mg/L	[29]
T	27	C	[29]
**Calculated rates**			
Gv	5.73	L/d	Calculated (see Table S1)
k1	5.93×10^{-1}	L/kg/d	Calculated (see Table S1)
k2	8.11×10^{-3}	d^{-1}	Calculated (see Table S1)
kg	7.24×10^{-3}	d^{-1}	Calculated[1]
**Bioconcentration factor**			
Log BCF (Model)	0.587	L/kg	k1 / (k2 + kg)
Log BCF (Obs)	0.591	L/kg	[29] (see Table S6c)
Model Bias	**1.01**		

a – This is the same method that was used for PFAAs. Uptake rates were reported separately for different organs, so a whole-body uptake rate was estimated using a calculation analogous to the one used to calculate a whole-body BCF (Table S6c), using the same organ volume distribution shown in Table S6c.

Bioconcentration factors for HFPO-DA were reported for the carcass, fillet, plasma, and liver. A whole-body concentration factor was calculated based on estimated organ sizes, as estimated for the fish modeled by Ng & Hungerbuhler[17], and assuming approximately equal densities (1 g/mL) across organs. Details of this calculation are shown in the table below.

Table S6c. HFPO-DA whole body BCF estimation

Tissue	Observed BCF[29]	Organ volume (mL)[a]	Organ % fraction of whole body[a]
Fillet	2.200	3.7	46.25%
Plasma	2.967	0.017[b]	2.06%
Liver	0.262	0.091	1.14%
Carcass	5.583		50.55%[c]
**Whole Body BCF[d]**	**3.904**		
**Whole Body logBCF[d]**	**0.591**		

a – Compiled by Ng & Hungerbuhler[17], Table S1 for an 8 g fish
b – Assumes blood plasma is approximately 55% of whole blood volume
c – Calculated as the remaining fraction
d – Calculated as a volume-weighted average

Section 7. Estimation of fish protein content

Albumin volume % calculation

1. *Calculate whole blood volume percent in fish* – given the volumes and body weight percent for each organ tissue modeled in Ng & Hungerbuhler[17] (Table S1, assuming a similar density between organs), the volume of a whole fish is approximately 7.97 mL. Given a blood volume of 0.3 mL, blood is approximately 3.8% of the total body volume, similar to other literature values (Karlssonnorrgren et al. 1985 as cited in Shi et al.[30]).

2. *Estimate albumin content in whole blood* – Albumin content in fish blood can be estimated based on estimated albumin content in human blood. Human blood has an albumin concentration of about 30-50 g/L and a density of about 1060 g/L; therefore, albumin makes up approximately 3.3-4.7% of human blood, Albumin is estimated to make up about 65% of all proteins in human blood, but varies from 0-60% across the several fish species that have been measured[13,14]. In rainbow trout, the species studied in lab-based evaluation dataset in the current study, the albumin concentration has been measured at about 13.8 +/- 0.5 g/L, or about 38% of total protein (35.9 +/- 1.3 g/L)[31]; similar albumin:total protein ratios have been observed in a range of species, including rainbow trout, channel catfish, tilapia, striped bass, salmon, three species of carp[14,32,33]. Assuming a similar ratio between total protein content and blood density in humans and fish, we would expect the albumin contribution to whole blood for these common fish species to be about two-thirds of the value estimated for human blood.

   Albumin content in fish can be highly variable among species, and or the same species under varying environmental conditions or life stages. It is also possible that some species that do not express albumin can instead express other proteins serving similar functions, and that can also bind PFAAs. For example, Zhong et al.[12] report PFAA binding affinities for "fish blood proteins" in common carp, which have been reported not to express albumin[13], but do express lipoproteins that serve similar functions. The lipoprotein:protein ratio measured in one carp study was also in the 40% range, although it was noted that the lipoprotein concentration varied seasonally and based on diet[34]. This variability contributes to making albumin volume percent, or binding protein percent more generally, one of the more uncertain parameters in the current model.

3. *Blood albumin % in whole body* – Using an approximate maximum albumin content in whole blood of 3% and a blood volume percent of 3.8%, we calculate that albumin in the blood is about 0.11% of total body weight.

4. *Calculate albumin % distribution in blood* – Based on values used to parameterize the Ng & Hungerbuhler[17] PBTK bioconcentration model for PFAAs, the estimated fraction of albumin in whole blood is 75.5% (see Table S6a). In contrast, based

on a broader literature review, Ng & Hungerbuhler[35] state that between "30-40% of the total albumin pool in an organism is generally believed to be present in the plasma, with the remainder distributed in the extravascular fraction." While this estimate is not specifically made for fish, it would include albumin present in other tissue compartments not included in the Ng & Hungerbuhler model.

Table S7a. Calculation of protein content in blood and other tissues in a model fish. Protein concentration and tissue volumes were taken from Ng & Hungerbuhler[17] (Tables S1, S2 & S5). These values were used to parameterize a PBTK model that was evaluated against the same laboratory bioconcentration study data used in the current study.

	Ng & Hungerbuhler 2013 model values				
	Protein Concentration (mmol/L)	Tissue Volume (mL)	Protein content (nmoL)	% of total protein content (albumin or FABP)	% of total protein content (albumin + FABP)
**Albumin**					
Blood	0.2	0.3	60	75.5%	71.4%
Liver Fluid	0.1	0.026	2.6	3.3%	
Kidney Fluid	0.1	0.045	4.5	5.7%	
Muscle Fluid	0.06	0.20	12	5.1%	
Adipose Fluid	0.03	0.012	0.36	0.5%	
Total Albumin			79.46	100%	
**Fatty Acid Binding Protein**					
Liver Tissue	0.05	0.091	4.55	100%	5.4%
**Albumin + Fatty Acid Binding Protein**					
Total Albumin + L-FABP			84.01	100%	

5. *Total albumin % in whole body* – If albumin in blood is about 75.5% of total albumin in the body (60 nmoL / 79.46 nmoL = 75.5%), then we can calculate that all albumin in the body makes up about 0.15% of total body weight (albumin in blood = 0.11% of total body weight; 0.11% / 0.755 = 0.146%). On the other extreme, if we assume blood albumin is only 30% of total albumin, then we calculate a total albumin volume percent in the body of about 0.38% (0.11% / 0.30 = 0.38%).

In the current model, we use an albumin protein volume percent of 0.3% to represent an upper end estimate of protein contribution to PFAA partitioning to tissues. This is similar to the estimated blood protein volume content of 0.27% independently estimated by Shi et al.[30] (adapted from Nichols et al. 1990).

Liver fatty acid binding protein

As discussed in Section 3, L-FABP was not included in this model because its estimated total contribution to protein binding is negligible compared to uncertainty in the albumin volume percent parameter. Protein binding studies using mammalian proteins suggest that PFAAs bind more weakly to L-FABP than albumin, but in this calculation we conservatively assume similar partitioning to both proteins. Based on the protein abundance estimated in Table S7a, the addition of L-FABP increases the total protein binding (albumin + L-FABP) pool by approximately 6% (4.55 nmoL / 84.01 nmoL = 0.057). This in turn increases our estimate of the fraction of total body volume made up of binding proteins by 6%, from 0.15%-0.38% (albumin only) to 0.16%-0.40%. Our current estimate of binding protein content in the model (0.3%) remains well within this revised range of albumin + L-FABP content. Therefore, while L-FABP is not explicitly included as a separate compartment in this model, it could be considered accounted for within the compartment currently attributed to albumin proteins alone.

Section 8. Renal elimination rate calculation

Table S8. Renal elimination rate calculation

	C6 PFSA	C8 PFSA	C8 PFCA	C9 PFCA	C10 PFCA	C11 PFCA	Reference
Renal clearance (s^{-1})	0.023	0.050	0.029	0.050	0.049	0.062	[17,36]
Renal reabsorption (s^{-1})	0.004	0.037	0.014	0.037	0.042	0.059	[17,36]
Renal clearance-to-reabsorption ratio	5.88[a]	1.35[a]	2.08	1.35[a]	1.17	1.05	
Renal-to-total (renal + branchial) elimination ratio		0.21	0.92				[37,38]
**Calculate renal elimination**							
Branchial elimination (d^{-1})	0.003	0.051	0.006	0.017[b]	0.031	0.058	[3], This study
Renal elimination (d^{-1})	0.315[d]	0.014[c]	0.069[c]	0.014 [d]	0.007 [d]	0 [d]	This study (Figure S7)
Renal-to-total elimination ratio	0.99	0.21	0.92	0.44	0.19	0	This study

a – Values for PFSAs are based on renal clearance and reabsorption rates calculated for the same perfluorinated chain-length PFCA. Values for the C9 PFCA are not reported in Ref. 1 but are reported for C8 PFSA and are assumed to be the same for these two compounds.
b – Calculated using uptake rate from a linear regression between PFCA chain length and uptake rate
c – Calculated using measured renal-to-total elimination ratio and modeled branchial elimination rates
d – Calculated using linear regression (see Figure S7)’

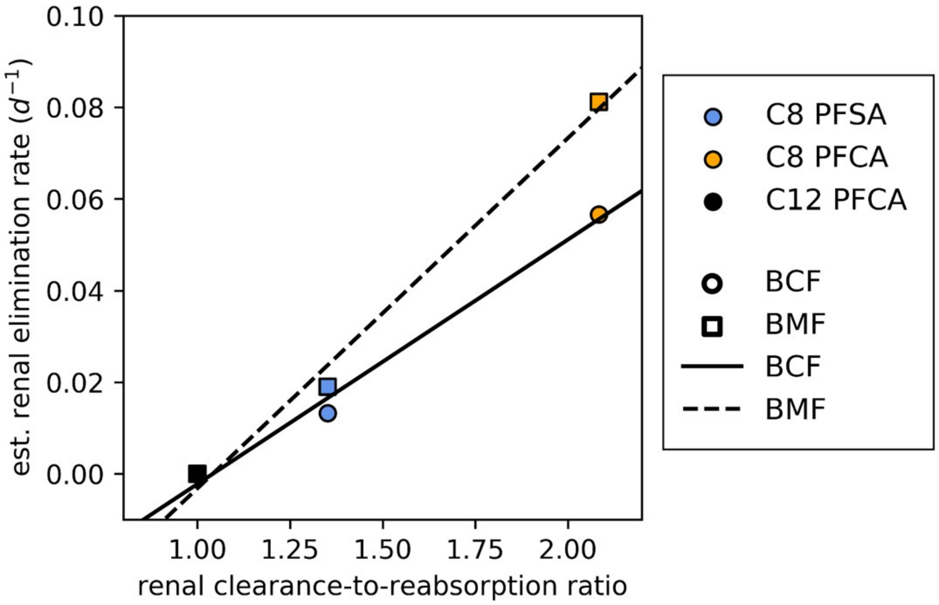

Figure S8. Estimation of renal elimination rates from linear regression between renal elimination rate and renal clearance-to-reabsorption ratio for the C8 PFSA, C8 PFCA and C12 PFCA. Linear regressions were calculated separately for the bioconcentration (circles, solid line, $R^2 = 0.98$) and biomagnification (squares, dotted line, $R^2 = 0.98$) datasets. For the remaining PFAAs, renal elimination rates for fish in the bioconcentration and biomagnification studies were estimated from the linear regression relationships based on renal clearance-to-reabsorption ratios calculated from data reported by Weaver et al.[36] and Ng & Hungerbuhler[35]. Further details of this calculation are described in Table S7 and the main text.

Section 9. Sensitivity Analysis

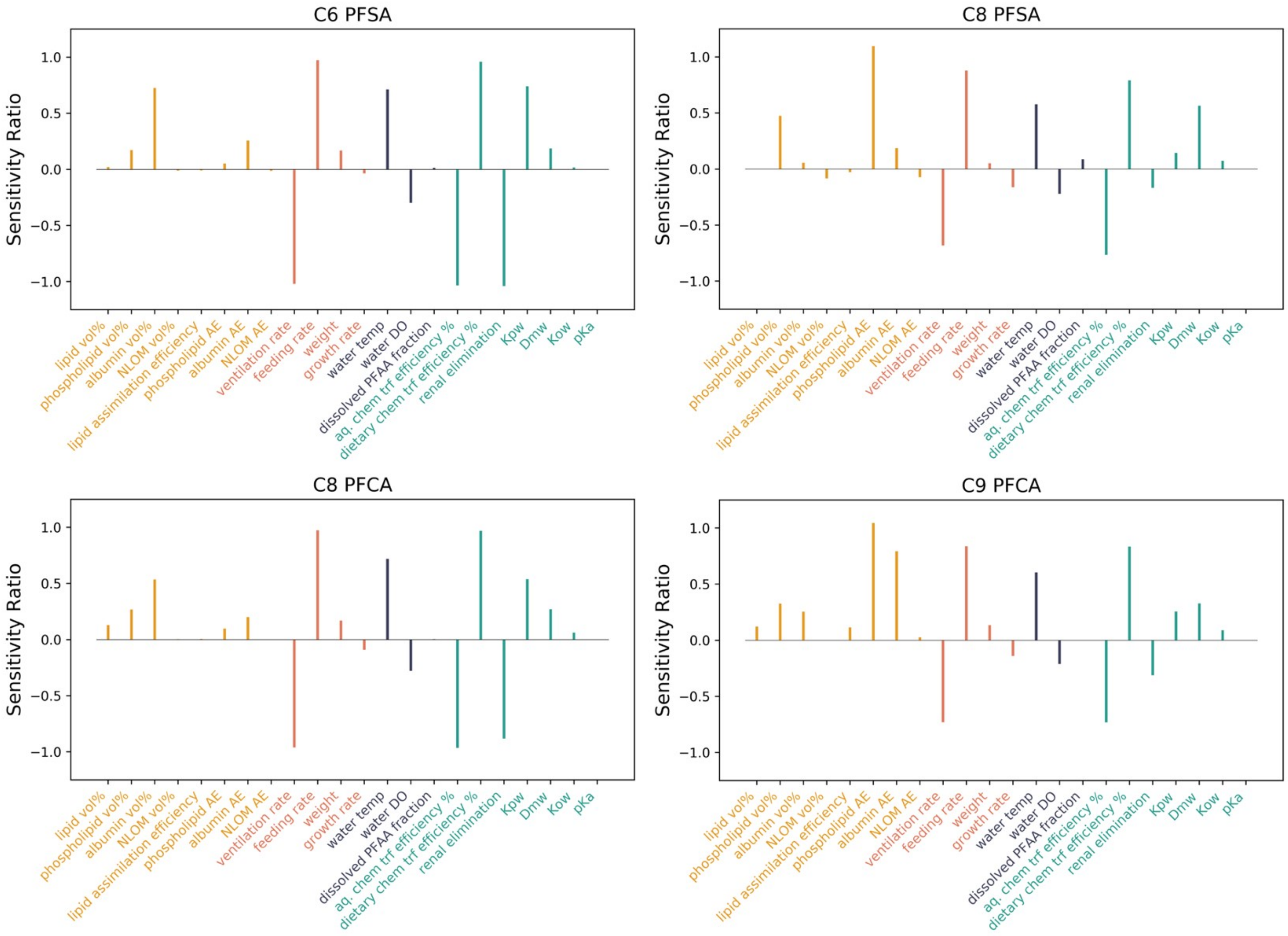

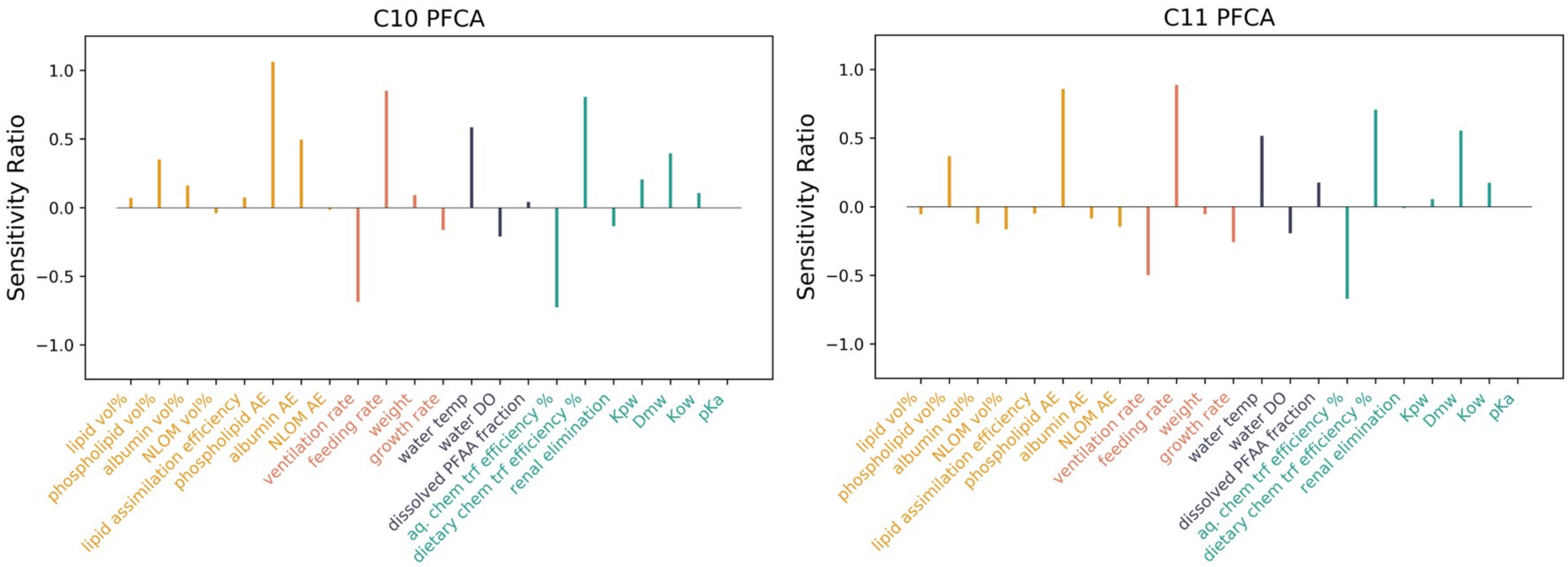

Figure S9. Sensitivity ratios for key parameters calculated using a representative fish species (Sole) from the Gironde Estuary system. Parameters evaluated include organism parameters (yellow; tissue composition and assimilation efficiencies), bioenergetics (orange), environmental characteristics (purple), and chemical-specific physiochemical properties or mechanisms (green).

Section 10. Influence of dietary exposure on modeled food web concentrations

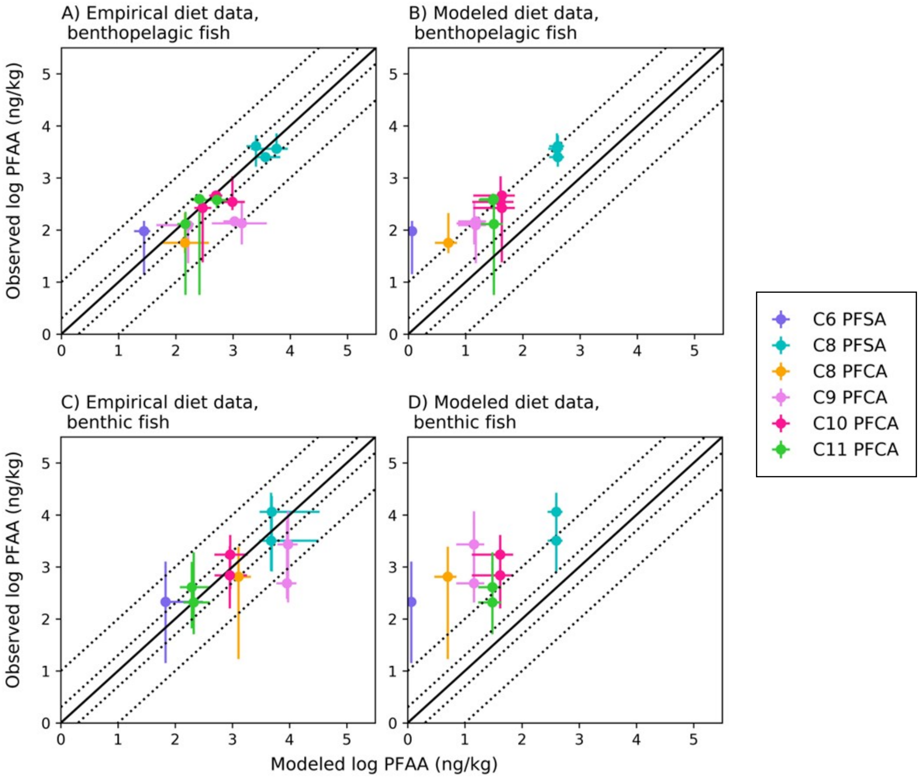

Figure S10. Comparison of model-predicted PFAA concentrations for both benthopelagic and fully benthic fish from the Gironde Estuary, France, simulated using observed prey data or no dietary uptake. The solid black line represents a 1:1 line (perfect agreement), while the dotted lines represent a factor of two and a factor of 10 difference between modeled and observed values. Circles show modeled values based on median water/sediment/prey concentrations and error bars represent minimum and maximum reported exposure concentrations. Observed variability likely includes variability not included in the model, such as variability in organism or environmental parameters. Results show that dietary uptake plays an important role in total exposures for all fish, but has greatest influence in the benthic food web.

References

(1) Arnot, J. A.; Gobas, F. A. P. C. A Food Web Bioaccumulation Model for Organic Chemicals in Aquatic Ecosystems. *Environ. Toxicol. Chem.* **2004**, *23* (10), 2343–2355. https://doi.org/10.1897/03-438.

(2) Armitage, J. M.; Arnot, J. A.; Wania, F.; Mackay, D. Development and Evaluation of a Mechanistic Bioconcentration Model for Ionogenic Organic Chemicals in Fish. *Environ. Toxicol. Chem.* **2013**, *32* (1), 115–128. https://doi.org/10.1002/etc.2020.

(3) Martin, J. W.; Mabury, S. A.; Solomon, K. R.; Muir, D. C. G. Bioconcentration and Tissue Distribution of Perfluorinated Acids in Rainbow Trout (*Oncorhynchus Mykiss*). *Environ. Toxicol. Chem.* **2003**, *22* (1), 196–204. https://doi.org/10.1002/etc.5620220126.

(4) Ebert, A.; Allendorf, F.; Berger, U.; Goss, K.-U.; Ulrich, N. Membrane/Water Partitioning and Permeabilities of Perfluoroalkyl Acids and Four of Their Alternatives and the Effects on Toxicokinetic Behavior. *Environ. Sci. Technol.* **2020**, acs.est.0c00175. https://doi.org/10.1021/acs.est.0c00175.

(5) Martin, J. W.; Mabury, S. A.; Solomon, K. R.; Muir, D. C. G. Dietary Accumulation of Perfluorinated Acids in Juvenile Rainbow Trout (*Oncorhynchus Mykiss*). *Environ. Toxicol. Chem.* **2003**, *22* (1), 189–195. https://doi.org/10.1002/etc.5620220125.

(6) Goeritz, I.; Falk, S.; Stahl, T.; Schäfers, C.; Schlechtriem, C. Biomagnification and Tissue Distribution of Perfluoroalkyl Substances (PFASs) in Market-Size Rainbow Trout (Oncorhynchus Mykiss). *Environ. Toxicol. Chem.* **2013**, *32* (9), 2078–2088. https://doi.org/10.1002/etc.2279.

(7) Droge, S. T. J. Membrane–Water Partition Coefficients to Aid Risk Assessment of Perfluoroalkyl Anions and Alkyl Sulfates. *Environ. Sci. Technol.* **2019**, *53* (2), 760–770. https://doi.org/10.1021/acs.est.8b05052.

(8) MacManus-Spencer, L. A.; Tse, M. L.; Hebert, P. C.; Bischel, H. N.; Luthy, R. G. Binding of Perfluorocarboxylates to Serum Albumin: A Comparison of Analytical Methods. *Anal. Chem.* **2010**, *82* (3), 974–981. https://doi.org/10.1021/ac902238u.

(9) Bischel, H. N.; MacManus-Spencer, L. A.; Zhang, C.; Luthy, R. G. Strong Associations of Short-Chain Perfluoroalkyl Acids with Serum Albumin and Investigation of Binding Mechanisms. *Environ. Toxicol. Chem.* **2011**, *30* (11), 2423–2430. https://doi.org/10.1002/etc.647.

(10) Allendorf, F.; Berger, U.; Goss, K.-U.; Ulrich, N. Partition Coefficients of Four Perfluoroalkyl Acid Alternatives between Bovine Serum Albumin (BSA) and Water in Comparison to Ten Classical Perfluoroalkyl Acids. *Environ. Sci. Process. Impacts* **2019**, *21* (11), 1852–1863. https://doi.org/10.1039/C9EM00290A.

(11) Alesio, J. L.; Slitt, A.; Bothun, G. D. Critical New Insights into the Binding of Poly- and Perfluoroalkyl Substances (PFAS) to Albumin Protein. *Chemosphere* **2022**, *287*, 131979. https://doi.org/10.1016/j.chemosphere.2021.131979.

(12) Zhong, W.; Zhang, L.; Cui, Y.; Chen, M.; Zhu, L. Probing Mechanisms for Bioaccumulation of Perfluoroalkyl Acids in Carp (Cyprinus Carpio): Impacts of Protein Binding Affinities and Elimination Pathways. *Sci. Total Environ.* **2019**, *647*, 992–999. https://doi.org/10.1016/j.scitotenv.2018.08.099.
(13) Enerstvedt, K. S.; Sydnes, M. O.; Pampanin, D. M. Identification of an Albumin-like Protein in Plasma of Atlantic Cod (Gadus Morhua) and Its Biomarker Potential for PAH Contamination. *Heliyon* **2017**, *3* (8). https://doi.org/10.1016/j.heliyon.2017.e00367.
(14) Sandnes, K.; Lie, O.; Waagbo, R. Normal Ranges of Some Blood Chemistry Parameters in Adult Farmed Atlantic Salmon, Salmo Salar. *J. Fish Biol.* **1988**, *32* (1), 129–136. https://doi.org/10.1111/j.1095-8649.1988.tb05341.x.
(15) Zhang, L.; Ren, X.-M.; Guo, L.-H. Structure-Based Investigation on the Interaction of Perfluorinated Compounds with Human Liver Fatty Acid Binding Protein. *Environ. Sci. Technol.* **2013**, *47* (19), 11293–11301. https://doi.org/10.1021/es4026722.
(16) Chen, Y.-M.; Guo, L.-H. Fluorescence Study on Site-Specific Binding of Perfluoroalkyl Acids to Human Serum Albumin. *Arch. Toxicol.* **2008**, *83* (3), 255. https://doi.org/10.1007/s00204-008-0359-x.
(17) Ng, C. A.; Hungerbühler, K. Bioconcentration of Perfluorinated Alkyl Acids: How Important Is Specific Binding? *Environ. Sci. Technol.* **2013**, *47* (13), 7214–7223. https://doi.org/10.1021/es400981a.
(18) Saarikoski, J.; Lindstrm, R.; Tyynela, M.; Viluksela, M. 0 - OLD Factors Affecting the Absorption of Phenolics and Carboxylic Acids in the Guppy (Poecilia Reficulata). 16.
(19) Yang, C.-H.; Glover, K. P.; Han, X. Characterization of Cellular Uptake of Perfluorooctanoate via Organic Anion-Transporting Polypeptide 1A2, Organic Anion Transporter 4, and Urate Transporter 1 for Their Potential Roles in Mediating Human Renal Reabsorption of Perfluorocarboxylates. *Toxicol. Sci.* **2010**, *117* (2), 294–302. https://doi.org/10.1093/toxsci/kfq219.
(20) Armitage, J. M.; Erickson, R. J.; Luckenbach, T.; Ng, C. A.; Prosser, R. S.; Arnot, J. A.; Schirmer, K.; Nichols, J. W. Assessing the Bioaccumulation Potential of Ionizable Organic Compounds: Current Knowledge and Research Priorities. *Environ. Toxicol. Chem.* **2017**, *36* (4), 882–897. https://doi.org/10.1002/etc.3680.
(21) Lorber, M.; Egeghy, P. P. Simple Intake and Pharmacokinetic Modeling to Characterize Exposure of Americans to Perfluoroctanoic Acid, PFOA. *Environ. Sci. Technol.* **2011**, *45* (19), 8006–8014. https://doi.org/10.1021/es103718h.
(22) Egeghy, P. P.; Lorber, M. An Assessment of the Exposure of Americans to Perfluorooctane Sulfonate: A Comparison of Estimated Intake with Values Inferred from NHANES Data. *J. Expo. Sci. Environ. Epidemiol.* **2011**, *21* (2), 150–168. https://doi.org/10.1038/jes.2009.73.
(23) Munoz, G.; Budzinski, H.; Babut, M.; Lobry, J.; Selleslagh, J.; Tapie, N.; Labadie, P. Temporal Variations of Perfluoroalkyl Substances Partitioning between Surface Water, Suspended Sediment, and Biota in a Macrotidal Estuary. *Chemosphere* **2019**, *233*, 319–326. https://doi.org/10.1016/j.chemosphere.2019.05.281.

(24) Allendorf, F.; Goss, K.-U.; Ulrich, N. Estimating the Equilibrium Distribution of Perfluoroalkyl Acids and 4 of Their Alternatives in Mammals. *Environ. Toxicol. Chem.* **2021**, *40* (3), 910–920. https://doi.org/10.1002/etc.4954.

(25) US EPA. *Estimation Programs Interface Suite for Microsoft Windows*; United States Environmental Protection Agency: Washington, DC, USA, 2022.

(26) Liu, W.; Yang, J.; Li, J.; Zhang, J.; Zhao, J.; Yu, D.; Xu, Y.; He, X.; Zhang, X. Toxicokinetics and Persistent Thyroid Hormone Disrupting Effects of Chronic Developmental Exposure to Chlorinated Polyfluorinated Ether Sulfonate in Chinese Rare Minnow. *Environ. Pollut.* **2020**, *263*, 114491. https://doi.org/10.1016/j.envpol.2020.114491.

(27) Xin, J.; Yan, S.; Hong, X.; Zhang, H.; Zha, J. Environmentally Relevant Concentrations of Carbamazepine Induced Lipid Metabolism Disorder of Chinese Rare Minnow (Gobiocypris Rarus) in a Gender-Specific Pattern. *Chemosphere* **2021**, *265*, 129080. https://doi.org/10.1016/j.chemosphere.2020.129080.

(28) Xin, J.; Yan, S.; Hong, X.; Zhang, H.; Zha, J. Environmentally Relevant Concentrations of Clozapine Induced Lipotoxicity and Gut Microbiota Dysbiosis in Chinese Rare Minnow (Gobiocypris Rarus). *Environ. Pollut.* **2021**, *286*, 117298. https://doi.org/10.1016/j.envpol.2021.117298.

(29) Siddiqui, S.; Fitzwater, M.; Scarpa, J.; Conkle, J. L. Comparison of Bioconcentration and Kinetics of GenX in Tilapia Oreochromis Mossambicus in Fresh and Brackish Water. *Chemosphere* **2022**, *287*, 132289. https://doi.org/10.1016/j.chemosphere.2021.132289.

(30) Shi, Y.; Vestergren, R.; Nost, T. H.; Zhou, Z.; Cai, Y. Probing the Differential Tissue Distribution and Bioaccumulation Behavior of Per- and Polyfluoroalkyl Substances of Varying Chain-Lengths, Isomeric Structures and Functional Groups in Crucian Carp. *Environ. Sci. Technol.* **2018**, *52* (8), 4592–4600. https://doi.org/10.1021/acs.est.7b06128.

(31) Manera, M.; Britti, D. Assessment of Blood Chemistry Normal Ranges in Rainbow Trout. *J. Fish Biol.* **2006**, *69* (5), 1427–1434. https://doi.org/10.1111/j.1095-8649.2006.01205.x.

(32) Zhang, W.; Liang, G.; Wu, L.; Tuo, X.; Wang, W.; Chen, J.; Xie, P. Why Mammals More Susceptible to the Hepatotoxic Microcystins than Fish: Evidences from Plasma and Albumin Protein Binding through Equilibrium Dialysis. *Ecotoxicology* **2013**, *22* (6), 1012–1019. https://doi.org/10.1007/s10646-013-1086-5.

(33) Hrubec, T. C.; Smith, S. A. Differences between Plasma and Serum Samples for the Evaluation of Blood Chemistry Values in Rainbow Trout, Channel Catfish, Hybrid Tilapias, and Hybrid Striped Bass. *J. Aquat. Anim. Health* **1999**, *11* (2), 116–122. https://doi.org/10.1577/1548-8667(1999)011<0116:DBPASS>2.0.CO;2.

(34) Nakagawa, H.; Kayama, M.; Asakawa, S. Biochemical Studies on Carp Plasma Protein. I. Isolation and Nature of an Albumin. *NIPPON SUISAN GAKKAISHI* **1976**, *42* (6), 677–685. https://doi.org/10.2331/suisan.42.677.

(35) Ng, C. A.; Hungerbühler, K. Bioaccumulation of Perfluorinated Alkyl Acids: Observations and Models. *Environ. Sci. Technol.* **2014**, *48* (9), 4637–4648. https://doi.org/10.1021/es404008g.

(36) Weaver, Y. M.; Ehresman, D. J.; Butenhoff, J. L.; Hagenbuch, B. Roles of Rat Renal Organic Anion Transporters in Transporting Perfluorinated Carboxylates with Different Chain Lengths. *Toxicol. Sci.* **2010**, *113* (2), 305–314. https://doi.org/10.1093/toxsci/kfp275.
(37) Consoer, D. M.; Hoffman, A. D.; Fitzsimmons, P. N.; Kosian, P. A.; Nichols, J. W. Toxicokinetics of Perfluorooctanoate (PFOA) in Rainbow Trout (Oncorhynchus Mykiss). *Aquat. Toxicol.* **2014**, *156*, 65–73. https://doi.org/10.1016/j.aquatox.2014.07.022.
(38) Consoer, D. M.; Hoffman, A. D.; Fitzsimmons, P. N.; Kosian, P. A.; Nichols, J. W. Toxicokinetics of Perfluorooctane Sulfonate in Rainbow Trout (*Oncorhynchus Mykiss*): Toxicokinetics of PFOS in Trout. *Environ. Toxicol. Chem.* **2016**, *35* (3), 717–727. https://doi.org/10.1002/etc.3230.
